# Learning dominant physical processes with data-driven balance models

**DOI:** 10.1038/s41467-021-21331-z

**Published:** 2021-02-15

**Authors:** Jared L. Callaham, James V. Koch, Bingni W. Brunton, J. Nathan Kutz, Steven L. Brunton

**Affiliations:** 1 Department of Mechanical Engineering, University of Washington, Seattle, WA USA; 2 Oden Institute for Computational & Engineering Sciences, University of Texas, Austin, TX USA; 3 Department of Biology, University of Washington, Seattle, WA USA; 4 Department of Applied Mathematics, University of Washington, Seattle, WA USA

**Keywords:** Applied mathematics, Computational science, Physics

## Abstract

Throughout the history of science, physics-based modeling has relied on judiciously approximating observed dynamics as a balance between a few dominant processes. However, this traditional approach is mathematically cumbersome and only applies in asymptotic regimes where there is a strict separation of scales in the physics. Here, we automate and generalize this approach to non-asymptotic regimes by introducing the idea of an equation space, in which different local balances appear as distinct subspace clusters. Unsupervised learning can then automatically identify regions where groups of terms may be neglected. We show that our data-driven balance models successfully delineate dominant balance physics in a much richer class of systems. In particular, this approach uncovers key mechanistic models in turbulence, combustion, nonlinear optics, geophysical fluids, and neuroscience.

## Abstract (web summary)

The dynamics of complex physical systems can be determined by the balance of a few dominant processes. Callaham et al. propose a machine learning approach for the identification of dominant regimes from experimental or numerical data with examples from turbulence, optics, neuroscience, and combustion.

## Introduction

It is well known across the engineering and physical sciences that persistent behaviors in complex systems are often determined by the balance of just a few dominant physical processes. This heuristic, which we refer to as dominant balance, has played a pivotal role in our study of systems as diverse as turbulence^1^, geophysical fluid dynamics^2,3^, and fiber optics^4^. It is also thought to play a role in the emerging fields of pattern formation^5–7^, wrinkling^8^, droplet formation^9^, and biofilm dynamics^10^. These balance relations provide reduced-order mechanistic models to approximate the full complexity of the system with a tractable subset of the physics.

The success of dominant balance models is particularly evident in the field of fluid mechanics. The Navier–Stokes equations describe behavior across a tremendous range of scales, from water droplets to supersonic aircraft and hurricanes. Thus, much of our progress has required simplifying the physics with nondimensional parameters that determine which terms are important for a specific problem. Perhaps the most well-known dimensionless quantity, the Reynolds number, embodies the balance between inertial and viscous forces in a fluid. Other nondimensional numbers capture the relative importance of inertial and Coriolis forces (Rossby number), inertia and buoyancy (Froude number), and thermal diffusion and convection (Rayleigh number), among dozens of other possible effects. In many situations, the magnitude of these coefficients determines the important mechanisms at work in a flow; conversely, they determine which mechanisms may be safely neglected. In geophysical flows, balance arguments bypass the incredible complexity of the ocean and atmosphere to identify driving mechanisms such as geostrophy, the thermal wind, Ekman layers, and western boundary currents^2,3^. Lighthill, one of the most influential fluid dynamicists of the 20th century, often relied on dominant balance arguments as physical motivation for his mathematical analyses^3,11^. Beyond fluid mechanics, asymptotic methods have been crucial in characterizing a diverse range of physical behavior.

Advanced statistical tools now allow analysis of the increasing wealth of data from modern experimental and numerical methods, but to date there is no direct link between these data and the powerful insights of asymptotic scaling analysis. This presents an exciting opportunity to leverage data-driven methods, which are driving changes in a wide range of fields, from control^12,13^ to turbulence modeling^14^, forecasting^15^, and extreme event prediction^16^. Although some studies have addressed the dominant balance problem by using expert knowledge to design application-specific clustering algorithms^17,18^ or a post hoc interpretation of unsupervised clustering in terms of dominant balance^19^, to our knowledge the general challenge of identifying local dominant balance regimes directly from data remains open.

**Fig. 1:**
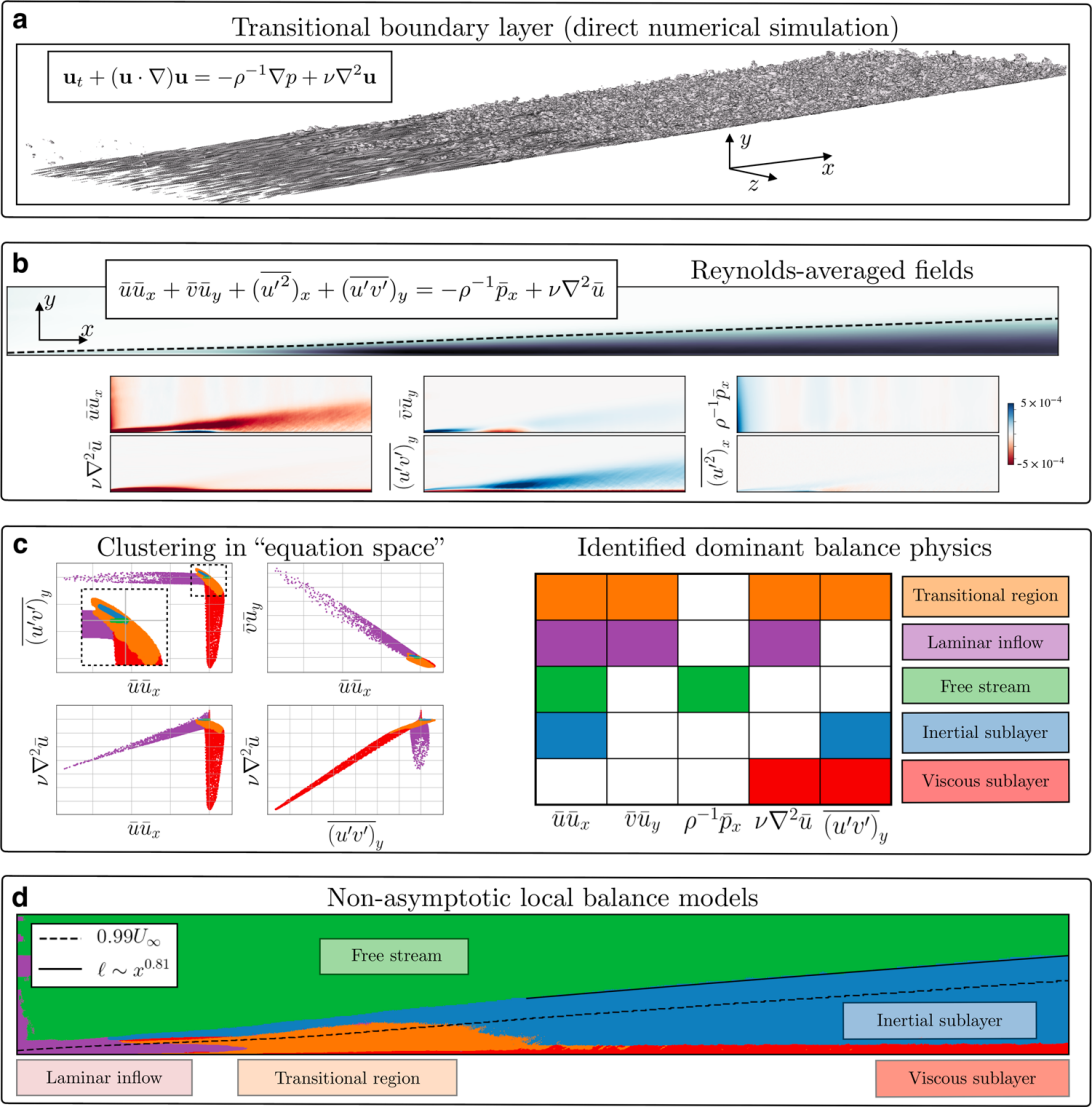
Schematic of the dominant balance identification procedure applied to a turbulent boundary layer. High-resolution direct numerical simulation results (**a**, visualized with a turbulent kinetic energy isosurface) are averaged to compute the terms in the Reynolds-averaged Navier–Stokes equations (**b**). The equation space representation of the field enables clustering and sparse approximation methods to extract the distinct geometrical structures in the six-dimensional space corresponding to dominant balance physics (**c**). Finally, the entire domain can be segmented according to these interpretable balance models, identifying distinct physical regimes (**d**). A curve fit to the wall-normal extent of the post-transition region of the identified inertial sublayer shows an approximate scaling of *ℓ* ~ *x*^0.81^, consistent with the theoretical prediction of *x*^4/5^ from boundary layer theory. The 99% free-stream velocity (*U*_∞_) contour is also shown for reference.

In this work, we develop a generalized data-driven method to identify dominant balance regimes in complex physical systems. Figure 1 demonstrates the method applied to fluid flow over a flat plate in transition to turbulence. We introduce a geometric perspective on dominant balance in which standard machine learning tools can automatically identify dominant physical processes. The geometric approach naturally links the analysis to the underlying equation so that the entire procedure can be easily interpreted and visualized. This data-driven method is designed to be applied in tandem with, rather than supplant, classical asymptotic analysis; the flexibility and generality of this combination extends balance modeling to a broader range of systems.

Our approach begins with a governing equation, which might be derived from fundamental physics (e.g., Maxwell’s equations or the Navier–Stokes equations) but could also result from a model discovery procedure^20–22^. These governing equations are physical models capable of describing a wide range of phenomena. However, it is well understood that the full complexity of such models is not always necessary to describe the local behavior of a system. In many regimes, the dynamics are governed by just a subset of the terms involved in the global description.

We introduce the idea of an equation space, where each coordinate is defined by one of the terms in the governing equation. Each term may be evaluated individually at any point in space and time, resulting in a vector with each entry corresponding to a term in the governing equation. We define a dominant balance regime as a region where the evolution equation is approximately satisfied by a subset of the original terms in the equation; the remaining terms may be safely neglected. When a point in the field is approximately in dominant balance, the equation space representation of the field will have near-zero entries corresponding to negligible terms. Clearly, the equation space representation of a field is not unique; a fluid flow might be represented by velocity, vorticity, or streamfunction, for example. The interpretation of the dominant physics therefore depends on the choice of an appropriate governing equation for the application.

Dominant balance physics thus has a natural geometric interpretation in equation space, allowing standard machine learning tools to automatically identify regions where groups of terms have negligible contributions to the local dynamics. From this perspective, a dominant balance regime is characterized by a cluster of points that have significant covariance in directions of equation space corresponding to active physical processes. The covariance structure of this cluster is sparse in the sense that there is weak variation in directions that represent the negligible terms. This corresponds to the mathematical condition that the governing equation is approximately satisfied by a subset of its terms in a local region.

While such dominant balance regimes might be identified by many possible algorithms, we choose to cluster the data using Gaussian mixture models (GMMs)^23^ and then extract a sparse approximation to the direction of maximum variance for each cluster using sparse principal components analysis (SPCA)^24^. We take the active terms in each cluster to be those that correspond to nonzero entries in the sparse approximation to the leading principal component.

In simple cases, this two-step GMM–SPCA procedure may be equivalent to applying a hard threshold, where a term is considered active if it exceeds some small value. However, our approach considers the local, relative importance of terms, whereas thresholding describes global, absolute importance. This distinction is important in multiscale systems where the scale of the dynamics varies significantly throughout the domain.

The data-driven approach to dominant balance analysis generalizes traditional methods in several critical directions. First, it does not rely on any explicit assumption of asymptotic scaling. Second, the clustering method yields pointwise estimates of the spatiotemporally local dominant balance not afforded by traditional scaling analysis in complex geometries. Third, while many dominant balance regimes have been proposed or assumed based on heuristic or intuitive arguments, this method provides an objective, reproducible approach to testing these hypotheses. Finally, the probabilistic Gaussian mixture modeling framework is fully compatible with the relative nature of dominant balance analysis, providing natural estimates of uncertainty in the identified balance (details in Supplementary Information).

## Results

**Fig. 2:**
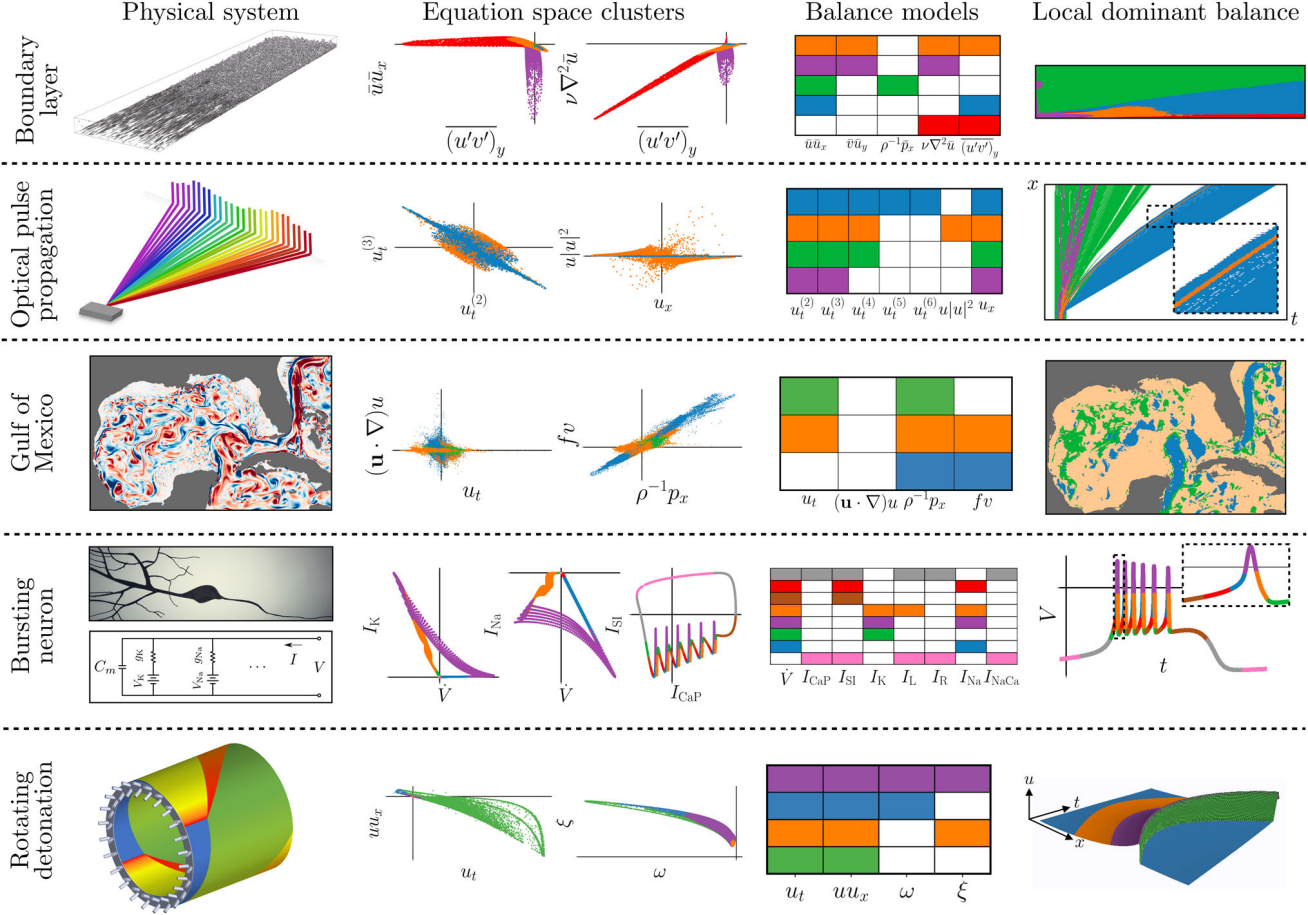
Dominant balance physics identified across a range of systems. For each case, a visualization of the system is shown on the left, followed by 2D views of the equation space colored by the identified balance relation, a key describing the active terms in each model, and the original field colored by the local balance. From top: a boundary layer in transition to turbulence, pulse propagation in an optical fiber, surface currents in the Gulf of Mexico, a Hodgkin–Huxley model for an intrinsically bursting neuron, and a simplified combustion model for a rotating detonation engine.

We apply the dominant balance identification method to a range of physics with varying complexity, as shown in Fig. 2: fluid flow in transition to turbulence; optical pulse propagation in supercontinuum generation; geostrophy in the Gulf of Mexico; a Hodgkin–Huxley-type model of a biological neuron; and a combustion analog for a rotating detonation engine (RDE). In each case, the results are consistent with classical scaling analyses or known physical behavior. While the results are well-established in the case of turbulence and geostrophy, we present the first objective dominant balance analysis of the supercontinuum generation, neuronal dynamics, and combustion analog systems. This demonstrates the ability to extract new physical insights and clarify misconceptions, for example, in the dominant balance that results in an emergent optical soliton. Detailed descriptions of the systems, including analytic scaling, are available in Supplementary Information.

### Boundary layer in transition to turbulence

One of the major breakthroughs in the study of fluid mechanics in the 20th century was the development of boundary layer theory^25^. In many practical applications, fluids can be treated as inviscid, but close to solid boundaries strong velocity gradients lead to significant viscous forces. Prandtl showed in 1904 that careful scaling analysis applied to the governing Navier–Stokes equations reveals distinct regimes where the behavior of the fluid is essentially determined by a small subset of the full equations. In turn, these balance relations can be used to derive powerful scaling laws such as the so-called law of the wall.

For an incompressible flow, the state variables are the velocity vector **u** = (*u*, *v*, *w*) and pressure *p*, with the fluid parameterized by density *ρ* and viscosity *ν*. After performing the Reynolds decomposition of the variables into mean and fluctuating components, e.g., $u({\bf{x}},t)=\bar{u}({\bf{x}})+u^{\prime} ({\bf{x}},t)$, the mean flow is determined by the Reynolds-averaged Navier–Stokes equations. For the streamwise mean velocity $\bar{u}$, the equation is

1 $$\bar{u}\frac{\partial \bar{u}}{\partial x}+\bar{v}\frac{\partial \bar{u}}{\partial y}={\rho }^{-1}\frac{\partial \bar{p}}{\partial x}+\nu {\nabla }^{2}\bar{u}-\frac{\partial }{\partial y}\overline{u^{\prime} v^{\prime} }-\frac{\partial }{\partial x}\overline{u{^{\prime} }^{2}}.$$

The terms on the left represent mean flow advection, while those on the right are the pressure gradient, viscosity, wall-normal Reynolds stress, and streamwise Reynolds stress, respectively.

We investigate the dominant balance physics of a boundary layer in transition to turbulence using data from a direct numerical simulation^18^. Figure 1 shows the equation space clusters and associated dominant balance models for the mean fields. Some sets of points have significantly reduced variance in certain directions of equation space, a strong signature of the dominant balance phenomenon. The method identifies regions corresponding to the viscous sublayer, inertial sublayer, and slightly perturbed free stream. It also identifies a region near the inlet characterized by a lack of Reynolds stresses, suggesting the mean profile here should be consistent with the laminar solution, as well as a transitional region between the laminar inflow region and fully developed turbulence downstream.

Dominant balance analysis is a starting point for many of the results of boundary layer theory, for instance, in making experimentally observable predictions for the profiles and scaling of wall turbulence^26,27^. Although we hope that data-driven balance identification will open new avenues of analysis, we can also use established results to examine the consistency of the proposed method. For example, the dominant length scale *ℓ* in the inertial sublayer is expected to depend on the streamwise coordinate *x* via a power law *ℓ* ~ *x*^4/5^ ^25^. It is not usually obvious how to extract a specific value of *ℓ* for which this scaling can be checked. However, as a rough proxy, we may consider the wall-normal coordinate at which the dominant balance changes from that of the inertial sublayer to the free stream. Figure 1 shows that the growth of the inertial sublayer thickness according to this definition closely agrees with the theoretical value.

### Nonlinear optical pulse propagation

**Fig. 3:**
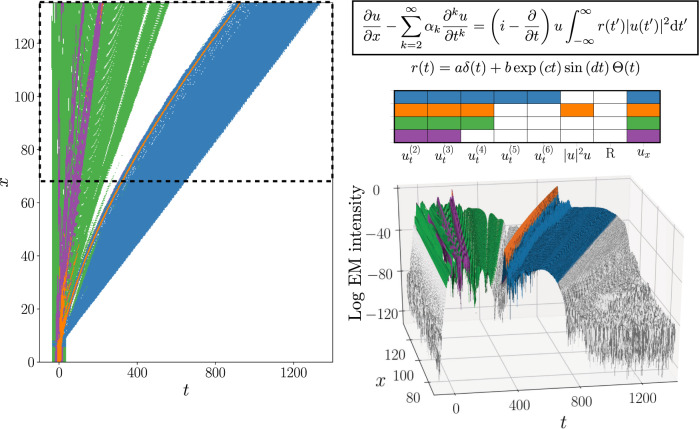
Nonlinear optical pulse propagation. The governing equations are derived from Maxwell’s equations in 1D with a nonlinear time-delayed polarization response. Soliton propagation is understood to be maintained primarily by a balance between low-order dispersion and the cubic Kerr nonlinearity (delta-function component of the right-hand side integral)^31^. Although most of the field is identified with various linear dispersion relations, the strongest soliton is associated with cubic nonlinearity and dispersive terms through fourth order.

Another important example of dominant balance arises in nonlinear optics, where the interplay of an intensity-dependent index of refraction with chromatic dispersion can generate localized optical solitons^28^. Figure 3 shows an example of a process known as supercontinuum generation, in which nonlinear processes act on a localized pulse of light to broaden the optical spectrum, stretching an initial 20–30 nm bandwidth to hundreds of nanometers. This is typically accomplished in microstructured optical fibers^29^. The governing equation in this case is derived from Maxwell’s wave equation in one dimension through the rotating wave and slowly varying envelope approximations^30^. The original PDE is linear and second order in a vacuum, but in order to handle complicated polarization responses in fibers the field is expanded about the frequency of the original pulse^4,31^. This center frequency expansion leads to a Taylor series expansion of the linear polarization response, and the Raman convolution integral describing a time-delayed nonlinear response. The resulting PDE, known as a generalized nonlinear Schrödinger equation (GNLSE), describes the evolution of the slowly varying complex envelope *u*(*x*,*t*) of the pulse. When nondimensionalized with soliton scalings^31^, the envelope equation is

2a $$\frac{\partial u}{\partial x}-\sum_{k = 2}^{\infty }{\alpha }_{k}\frac{{\partial }^{k}u}{\partial {t}^{k}}=\left(i-\frac{\partial }{\partial t}\right)u\int_{-\infty }^{\infty }r(t^{\prime} )| u(t^{\prime} ){| }^{2}{\rm{d}}t^{\prime}$$

2b $$r(t)=a\delta (t)+b\exp (ct)\sin (dt){{\Theta }}(t).$$

The various constants (*α*_*k*_, *a*, *b*, *c*, *d*) describe the polarization response and are determined empirically.

Although the spectral domain is often of practical interest for studies of supercontinuum generation, in the time domain the pulse exhibits soliton behavior, as shown in Fig. 3. To leading order, the soliton propagation is typically understood to be maintained by a balance between the second-order dispersion and the instantaneous part of the nonlinear response, or intensity-dependent index of refraction. That is, evaluating the delta-function component of the Raman kernel leads to the cubic Kerr nonlinearity. If only this cubic nonlinearity and second-order dispersion are retained, the dynamics reduce to the usual nonlinear Schrödinger equation

3 $$i\frac{\partial u}{\partial x}+\frac{{\partial }^{2}u}{\partial {t}^{2}}+| u{| }^{2}u=0,$$

which admits a number of canonical optical soliton solutions which are commonly observed in many experimental settings^28^. Indeed, they are known to be persistent localized structures that emerge from initial conditions in optical fibers and/or mode-locked lasers.

Figure 3 shows the balance models obtained through the unsupervised balance identification procedure applied to regions of the field where the intensity is within 40 dB of the peak. Most of the domain is associated with various linear dispersion relations, corresponding to different propagation speeds. Only a narrow region containing the strongest soliton is identified with the instantaneous nonlinear response, suggesting that a linear description is sufficient for much of the domain. The standard nonlinear Schrödinger equation is never identified, although the soliton balance relation with cubic nonlinearity and fourth-order dispersion is consistent with standard truncation of the linear response at third or fourth order^4^. Interestingly, the full Raman time-delay response is never selected as an important term, although this is understood to be a critical mechanism for the initial scattering. Presumably, the GMM approach is not sensitive enough to detect this, possibly due to the clearly invalid underlying assumption of normally distributed data. To date, the ad hoc analysis of the various emergent structures have only qualitatively explained the origins of the observed phenomenon as the detailed numerical simulations do not disambiguate the contributions from the various terms of the high-fidelity model. The dominant balance identification allows for a quantitative assessment of the emergent physics, even when solitonic structures are embedded in a sea of dispersive linear radiation. Moreover, for the first time, the analysis suggests that the emergent solitons have a significant impact from fourth-order dispersion, as only recently discovered in pure-quartic soliton lasers^32^.

### Geostrophic balance in the Gulf of Mexico

One of the best examples of a field where balance modeling has been central to our understanding is geophysical fluid dynamics; a full description of ocean circulation requires not only the Navier–Stokes equations on a rotating Earth with complicated bathymetry but must also account for the effects of varying salinity, temperature, and pressure via a nonlinear equation of state. The ocean dynamics also couple to the atmosphere, geological processes, and solar forcing^2^. To a first approximation, surface currents can be modeled with the 2D incompressible Navier–Stokes equations on a rotating sphere

4 $$\frac{\partial {\bf{u}}}{\partial t}+({\bf{u}}\cdot \nabla ){\bf{u}}-f\hat{{\bf{k}}}\times {\bf{u}}=-\frac{1}{\rho }\nabla p$$

where *ρ* is the density (in general a function of temperature, pressure, and salinity), and *x* and *y* are defined in the zonal and meridional directions, respectively. The Coriolis parameter *f* is given in terms of the Earth’s angular velocity Ω and the latitude *ϕ* by $f={{\Omega }}\sin \phi$. Note that this equation already includes some approximations. Compressibility, vertical motions, and both molecular and turbulent viscosities are all ignored in this model. Nevertheless, these equations are a standard starting point for many analyses of large-scale ocean dynamics.

However, scaling analysis suggests that in many cases, further simplified versions of the governing equations are sufficient to describe the large-scale motions. Perhaps the most important model of this type is geostrophic balance, where the dominant balance is between the Coriolis forces and pressure gradient forces. Geostrophy is thought to describe most approximately steady large-scale currents^2^.

**Fig. 4:**
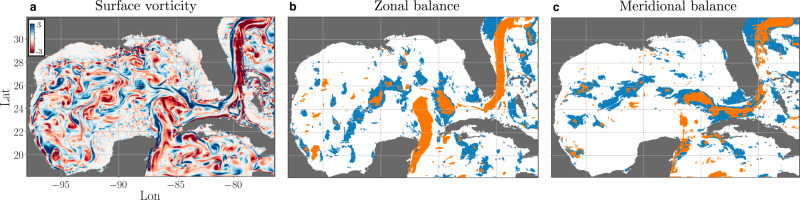
Circulation in the Gulf of Mexico. The flow is visualized with surface vorticity in units of s^−1^ (**a**) and identified balance models are shown for zonal (**b**) and meridional (**c**) dynamics. Orange regions are identified with the geostrophic balance, while the blue regions are time-varying in response to the pressure gradient and regions in white are associated with the linearized rotating Navier–Stokes equations.

We study the dominant balance of surface currents in the Gulf of Mexico using high-resolution HYCOM data. Our method identifies three regimes: geostrophic balance, a balance between acceleration and pressure gradients, and the linearized rotating Navier–Stokes equations (Fig. 4). The nonlinear advective term is not included in any of the models in this case, consistent with the common use of linearized equations to study wavelike motions. Geostrophic balance is primarily identified in regions corresponding to slowly varying, large-scale motions: the southern end of the Gulf Stream and the relatively stable current between Cuba and the Yucatàn Peninsula.

### Generalized Hodgkin–Huxley model

The dominant balance identification method can also be applied to systems that are not amenable to classic scaling analysis. For example, networks of biological neurons in an animal’s nervous systems communicate with each other through the propagation of electrical potentials. These all-or-nothing events, known as action potentials or spikes, are large deviations from the membrane electrical potential at rest. Spikes can travel without significant degradation down the length of a neuron’s axon, which may be meters long.

The celebrated Hodgkin–Huxley model for spiking neurons reproduces an action potential through a balance of currents from multiple ions, each of which moves through the cell’s membrane across specialized channels and pores at different phases of a spike^33^. These nonlinear differential equations were the first detailed biophysical model to quantitatively describe the dynamic activity of neurons, and they underpin decades of ongoing attempts to understand more complex properties of neuronal electrical excitability. Hodgkin and Huxley originally modeled three ionic currents: sodium, potassium, and a leak. The voltage dynamics of a single action potential can then be expressed as a system of four ordinary differential equations; the balance of currents in these equations reflects the biophysical mechanisms. Adding more ionic currents and modeling the interactive balance of their dynamics produces more complex spiking behavior.

In particular, here we consider a generalized Hodgkin–Huxley model with ten currents that simulates the intrinsically bursting pattern of spikes observed in the R15 neuron of the sea slug *Aplysia*^34^. The R15 neuron has been used to study the mechanisms underlying intrinsic bursting, where several action potentials are generated in rapid succession interspersed with relative quiet with constant inputs. Under space-clamp conditions where an entire axon cable is considered to be spatially uniform, the equation describing the time-evolution of membrane voltage *V* under applied external input *I*_stim_ is

5 $${C}_{M}\dot{V}=-\sum _{j}{I}_{j}+{I}_{\text{stim}},$$

where *C*_*M*_ is the membrane capacitance and *I*_*j*_ are each of the ionic currents in current per unit area due to the flow of ions into and out of the cell.

**Fig. 5:**
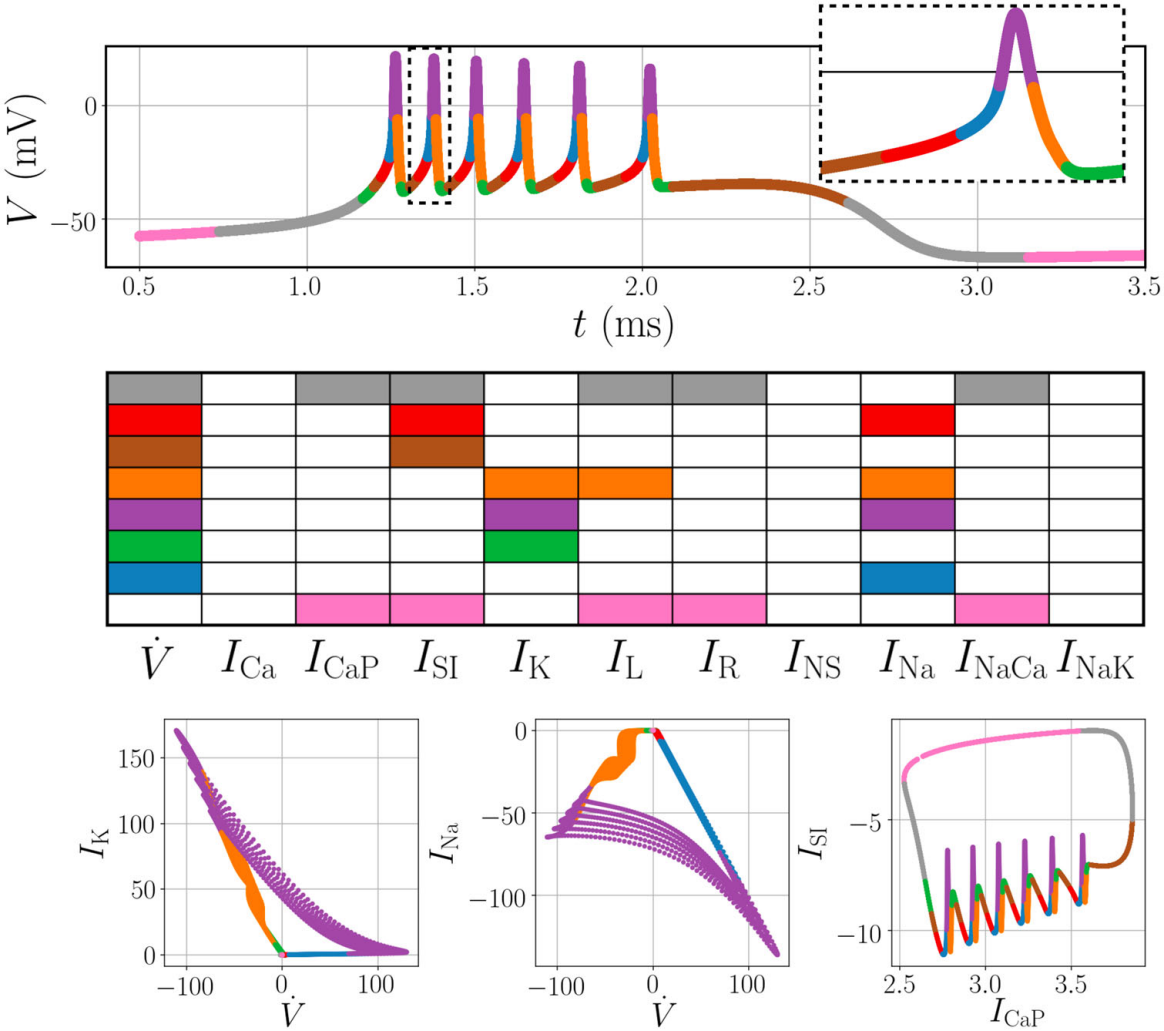
Model for an intrinsically bursting R15 *Aplysia* neuron. Dynamics in quiescent periods are characterized by currents related to calcium concentration (pink and gray), while the spiking dynamics are dominated by the classic sodium–potassium cycle.

Our dominant balance approach identifies several interpretable regimes of physics in the generalized Hodgkin–Huxley model that are largely consistent with known biophysics (Fig. 5). The addition of a set of calcium-dependent currents underly the slower oscillations between quiescence and excitable bursting, as evident in the slower limit cycle. In these clusters, the identified balance of ions is dominated by terms with strong calcium dependence (*I*_CaP_, *I*_SI_, and *I*_NaCa_). In contrast, the voltage during fast spikes is dominated by voltage-gated ionic currents. The rising part of each spike is mediated by activation of sodium channels, and the inward *I*_SI_ and *I*_Na_ increase voltage. The voltage peaks as the sodium channels deactivate and delayed rectifier potassium channels *I*_*K*_ activate. The exit of potassium from the cell decreases the voltage back toward the resting potential.

### RDE analog

**Fig. 6:**
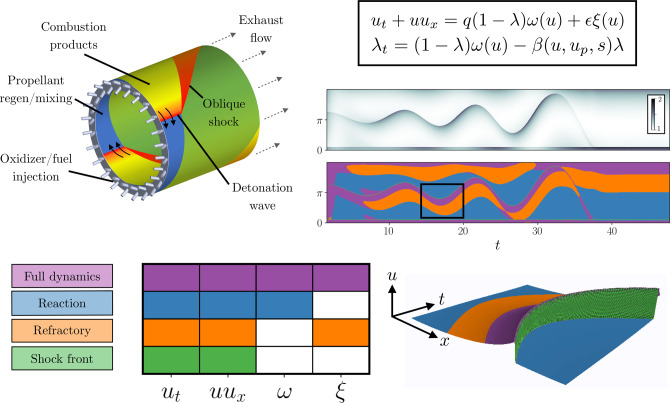
Model of combustion dynamics in a rotating detonation engine. The dynamics on the thin shock front are determined by the canonical Burgers balance (green), followed by activation of the gain and loss terms (purple). Following the combustion front, the balance transitions to the refractory exhaust-dominated period (orange). The rest of the domain is characterized by a combination of the Burgers dynamics with background energy input. All plots are visualized with the scalar state variable *u*(*x*,*t*).

Combustion systems are characterized by complex shifting balances between processes related to gas dynamics and chemical reactions, which typically unfold on dramatically different time scales. This suggests that the instantaneous local dynamics may be determined by a small subset of the relevant physics. For example, the RDE is a novel rocket engine combustor configuration that exploits the self-steepening properties of reactive compressible flows in confined, periodic geometries (such as an annular chamber, as depicted in Fig. 6) to form traveling detonation waves that persist in time. Globally, the stability of the traveling wave must therefore be maintained by a careful balance of energy input (combustion) and output (exhaust); locally, the balance is time and spatially varying in accordance with the nonlinear dynamics of the compressible gas.

The nonlinear dynamics of the annular RDE can be approximated with a surrogate Burgers–Majda model^35,36^. This detonation analog models the evolution of a quantity *u*(*x*, *t*) which is understood to be an abstract representation of an intensive property of the medium such as specific internal energy. These dynamics are supplemented with an evolution equation for a combustion progress variable, *λ*(*x*, *t*), which describes the balance of gain depletion and gain recovery

6a $${u}_{t}+u{u}_{x}=q(1-\lambda )\omega (u)+\epsilon \xi (u)$$

6b $${\lambda }_{t}=(1-\lambda )\omega (u)-\beta (u,{u}_{p},s)\lambda .$$

Here, *q* is the energy release associated with the reactive mixture, *ω*(*u*) is the submodel for kinetics, *ξ* is the submodel for exhaust (with a loss coefficient *ϵ*), and *β*(*u*, *u*_*p*_, *s*) is the injection and mixing submodel with parameters for an injection sensitivity cutoff *u*_*p*_ and overall timescale *s*. Details and further discussion are given in Supplementary Information. This model has been shown to qualitatively reproduce the nonlinear dynamics of the collection of detonation waves present in an RDE, including wave nucleation, destruction, modulation, and mode-locking.

Figure 6 shows a simulation of the system in the wave-attached reference frame with two traveling waves. Application of our dominant balance method identifies four distinct regions of physics. At the front of each wave is a thin region shaded in green. This region corresponds to the shock physics of the classic Burgers’ equation. For this region, *ω*(*u*) is approximately negligible, as the kinetics—an exponential function of *u*—are slow until *u* can activate *ω*(*u*). Eventually, an accumulation of *u* inside the domain is required before the nonlinear dissipation submodel—a quadratic function of *u*—becomes significant. This occurs in the purple shaded region, where the rate of energy input to the system (which is now slowed because of the (1 − *λ*) multiplier with 1 > *λ* > > 0) is of the same order as the dissipation term. Once *λ* ≈ 1, energy input becomes negligible, though dissipation is still significant; this region is shaded in orange. This region constitutes the refractory period behind the detonation wave where *u* and *λ* approach rest values. The remainder of the domain, shown in blue, is characterized by the balance of the nonlinear Burgers dynamics and autocatalytic background energy input.

## Discussion

In one guise or another, dominant balance analysis has played a major role in the development of our understanding of many complex systems. In this paper, we have proposed a method of identifying dominant balance regimes in an unsupervised manner directly from data. This approach leverages our understanding of the full physical complexity in the form of governing equations, but by using simple clustering and sparse approximation methods, we avoid any a priori assumptions about balance relations. Nevertheless, the method identifies dominant balance relationships that either recover classical scaling analysis (in the case of the boundary layer and Gulf of Mexico) or confirm arguments based on physical intuition (in the case of nonlinear optics, the Hodgkin–Huxley model, and the combustion analog).

The critical step in this process is the equation space perspective. By considering each term in the governing equation to describe a direction in this space, the dominant balance relations naturally manifest via restriction to subspaces, dramatically reducing variance in directions corresponding to negligible terms. This observation enables the GMM to identify clusters with variance in different directions, and the SPCA to extract sparse subspaces by finding directions with significantly nonzero variance. These machine learning tools are applied in a targeted and clearly motivated context, and the equation space perspective necessarily ties the output to underlying physics.

This data-driven approach has the same goal as traditional methods such as scaling analysis, but introduces several new features. It is a principled, objective approach that does not require the assumption of asymptotic parameter regimes, while providing an estimate of the locally active physical processes throughout domains with arbitrarily complex geometries. The proposed method retains the advantages of the classic approach, but generalizes to a range of disciplines to which traditional analysis cannot readily be applied.

Dominant balance analysis has historically been a critical tool for understanding local physical behavior in complex systems. Nonasymptotic data-driven methods could be used to better understand the behavior of more exotic dynamics such as non-Newtonian turbulence^37^ or to study important transitional behavior in cases where the asymptotics are already well known^38–40^. In the latter case, a clear understanding of the active mechanisms has proven crucial to successful control strategies^41,42^.

The existence of dominant balance limiting regimes even in complex nonlinear spatiotemporal systems is consistent with the observation that these systems can often be described with sparse representations in function space^21,22^. Building on this insight, we may even be able to identify local dominant balance behavior in spatiotemporal systems without clear governing equations, such as neuroscience, epidemiology, ecology, active fluids, and schooling. For example, the inclusion of spurious terms in the governing equation can be readily detected in the equation space representation (see Supplementary Information); in future work, this feature might be leveraged to identify local balance relations in the absence of global conservation equations. This approach thus stands to shed light on more exotic physical processes that have remained elusive to traditional analysis.

However, as with all applications of machine learning and data science methods to physical systems, a critical step in application to any system will be careful validation that the balance identification procedure reproduces the expected results. The dominant balance modeling approach described here is designed to build on, rather than circumvent, physical expertise. The study of dominant balance regimes has been foundational to our understanding of many complex systems; we hope that data-driven methods can integrate with this legacy to enable even wider applicability.

## Methods

The data-driven approach to dominant balance analysis is founded on the geometric perspective of equation space. This enables simple, widely available machine learning tools to identify spatiotemporal regions with different active physics. Details of the methods are given below.

### Equation space

A general evolution equation for the field *u*(*x*,*t*) on the domain $(x,t)\in {\mathcal{D}}$ can be written as

7 $${\mathcal{N}}(u)=\sum_{i = 1}^{K}{f}_{i}(u,{u}_{x},{u}_{xx},\ldots ,{u}_{t},\ldots \ )=0.$$

We represent the equation in implicit form both because it is the most general form and because it highlights the fundamental balance of the equation. At each point in (*x*, *t*) in space and time, each of the terms in the governing Eq. (7) may be evaluated at *u*(*x*, *t*), resulting in a *K*-dimensional vector **f** in equation space

8 $${\bf{f}}(x,t)={[{f}_{1}(u(x,t),\ldots )\cdots {f}_{K}(u(x,t),\ldots )]}^{T}.$$

Simulated or measured field data are typically discretized, so the domain is approximated by *N* spacetime points: ${\mathcal{D}}\approx \left\{{(x,t)}^{j}| j=1,2,\ldots ,N\right\}$. The field at each of these points corresponds to a point in equation space.

In many regimes, the dynamics are governed by just a subset of the terms involved in the global description. We define a dominant balance regime as a region ${\mathcal{R}}\subset {\mathcal{D}}$ where the evolution equation is approximately satisfied by a subset of *p* < *K* of the original terms in the equation, the remaining terms may be neglected. In this case, **f**(*x*, *t*) will have near-zero entries corresponding to negligible terms when $(x,t)\in {\mathcal{R}}$. Geometrically, the field is approximately restricted to *p* of the original *K* dimensions of the equation space, resulting in a subspace that is aligned with the active *p* terms.

### Gaussian mixture models

This geometric perspective on dominant balance physics leads naturally to segmentation via unsupervised clustering. For example, the GMM framework learns a probabilistic model by assuming the data are generated from a mixture of Gaussian distributions with different means and covariances^23^. The learned covariances for each cluster can then be interpreted in terms of active and inactive terms in the evolution equation. The *N* spacetime points in ${\mathcal{D}}$ are used to train a mixture model; the algorithm treats points from a dominant balance regime as if they were generated from a distribution with near-zero variance in the directions corresponding to negligible terms. Data beyond the original inputs can efficiently be assigned to a balance model using the trained GMM.

### Sparse principal components analysis

In practice, there is no reason to expect the points will even approximate a mixture of Gaussian distributions. We therefore expect that the number of clusters required to capture all of the relevant physics will exceed the number of distinct balance regimes, resulting in redundant clusters. Furthermore, there is some ambiguity in the interpretation of near-zero variance. We address both of these issues using SPCA^24^, which uses *ℓ*_1_ regularization to extract a sparse approximation to the leading principal component. If a cluster describes a dominant balance regime, it should be well-described by its direction of maximum variance. Moreover, this leading principal component should have many near-zero entries. We apply SPCA to the set of points in each GMM cluster and take the active terms in the cluster to be those which correspond to nonzero entries in the sparse approximation to the leading principal component.

### Dominant balance models

Each GMM cluster now has a sparse approximation to its leading principal component. Since the axes in equation space correspond directly to physical processes via the terms in the governing equation, we may interpret nonzero entries in the SPCA vector as active terms in the corresponding cluster. Different GMM clusters may have the same sparsity pattern, these are considered to be part of the same dominant balance regime. Points from all clusters with the same SPCA sparsity pattern are therefore combined into a single balance model (Fig. 1c and second column in Fig. 2). Once the equation space representation of the spatiotemporal data is fully grouped into balance models, the original domain can be segmented according to the dominant physical processes in each local region (Fig. 1d and last column in Fig. 2).

## Supplementary information

Supplementary Information

## Data Availability

The turbulent boundary layer data are openly available from the Johns Hopkins Turbulence Database^43^. Source code for simulating the GNLSE is available at http://www.scgbook.info. Surface current estimates in the Gulf of Mexico are from the HYCOM + NCODA global 1/25^∘^ reanalysis (Expt. 50.1) available at https://hycom.org. The detonation analog model was simulated with Clawpack^44^. Further information about the data sets and simulations are included in Supplementary Information.

## Code availability

All codes used to perform the analyses are available from the repository http://www.github.com/dynamicslab/dominant-balance^45^.
