# Supplementary material for: Learning dominant physical processes with data-driven balance models
Source: Nat Commun. 2021 Feb 15;12:1016. doi: 10.1038/s41467-021-21331-z (PMC7884409; doi:10.1038/s41467-021-21331-z)
Supplement: Supplementary file 1 — Supplementary Information [file 41467_2021_21331_MOESM1_ESM.pdf]

Supplementary information:
Learning dominant physical processes with data-driven balance models

Jared L. Callaham[1*], James V. Koch[2], Bingni W. Brunton[3],
J. Nathan Kutz[4], and Steven L. Brunton[1]

[1] Department of Mechanical Engineering, University of Washington, Seattle, WA 98195, United States
[2] Oden Institute for Computational & Engineering Sciences, University of Texas, Austin, TX 78712
[3] Department of Biology, University of Washington, Seattle, WA 98195, United States
[4] Department of Applied Mathematics, University of Washington, Seattle, WA 98195, United States

Supplementary Note 1 - Unsupervised dominant balance identification

In many fields of physics, painstaking analyses have produced models that are capable of describing a wide range of physical phenomena. However, it is well understood that the full complexity of such models is not always necessary to describe the local behavior of a system. We find that in many regimes the dynamics are governed by just a subset of the terms involved in the global description. A general evolution equation for the field $u(x,t)$ on the domain $(x,t) \in \mathcal{D}$ can be written as

$$\mathcal{N}(u) = \sum_{i=1}^{K} f_i(u, u_x, u_{xx}, \dots, u_t, \dots) = 0. \tag{1}$$

For example, the viscous Burgers' equation is

$$\mathcal{N}(u) = u_t + uu_x - \nu u_{xx} = 0 \tag{2}$$

We represent the equation in this implicit form rather than the typical $u_t = \mathcal{F}(u)$ form for two reasons. First, it includes arbitrary PDEs which are not easily expressed in the standard form, such as the generalized nonlinear Schödinger equation in Sec. . Second, this form highlights the fundamental balance of the equation; all terms must sum to zero. If some subset is are dominant, the rest must be relatively small.

Classically, this equation would be derived from fundamental physics (e.g. Maxwell's equations or the Navier-Stokes equations), but it could result from a model discovery procedure [1–3]. We assume the number of terms K is known, either from the physical model or as the result of a model selection procedure [4].

Proposed method

Consider an "equation space" where each coordinate is defined by one of the K terms in Eq. (1). At each point (x,t) in space and time, each of the K terms f_i in the governing equations (1) may be evaluated at $u(x,t)$, resulting in a vector $\mathbf{f} \in \mathbb{R}^K$:

$$\mathbf{f}(x,t) = \begin{bmatrix} f_1(u(x,t),\dots) & f_2(u(x,t),\dots) & \cdots & f_K(u(x,t),\dots) \end{bmatrix}^T. \tag{3}$$

By construction, $\mathbb{1}^T \mathbf{f}(x,t) = \mathcal{N}(u) = 0$ for all $(x,t) \in \mathcal{D}$. Simulated or measured field data is typically discretized, so the domain is approximated by N spacetime points: $\mathcal{D} \approx \left\{ (x,t)^j \mid j = 1, 2, \dots, N \right\}$. The field at each of these points corresponds to a point in equation space.

* Corresponding author (jc244@uw.edu)

Python code: github.com/dynamicslab/dominant-balance DOI:10.5281/zenodo.4428904

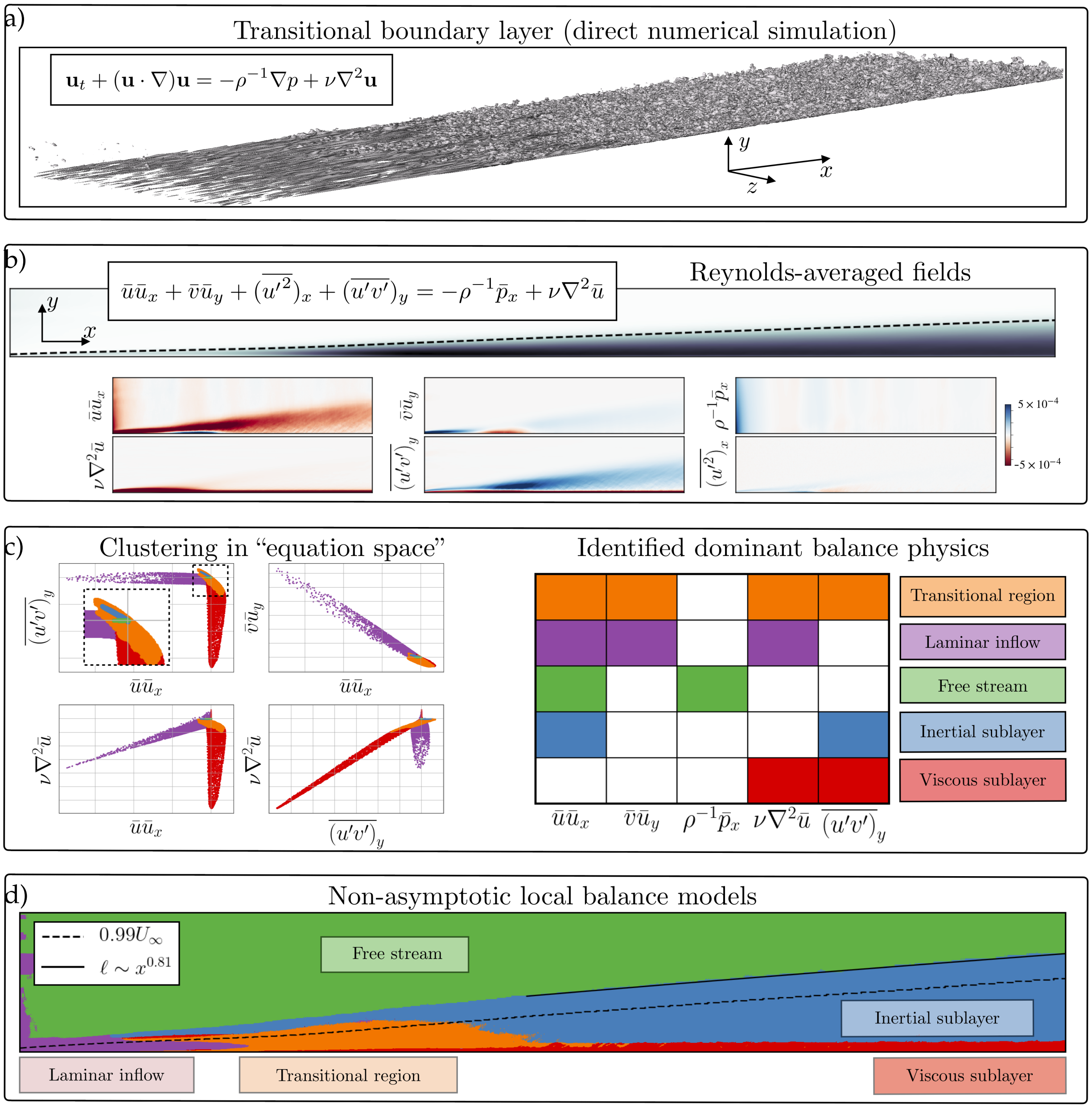

Supplmentary Figure 1: Schematic of the dominant balance identification procedure applied to a turbulent boundary layer. High-resolution direct numerical simulation results (a, visualized with a turbulent kinetic energy isosurface) are averaged to compute the Reynolds-averaged Navier-Stokes equations (b). The equation space representation of the field enables clustering and sparse approximation methods to extract the distinct geometrical structures in the six-dimensional space corresponding to dominant balance physics (c). Finally, the entire domain can be segmented according to these interpretable balance models, identifying distinct physical regimes (d). The equations and classical scaling analysis are discussed in Sec. .

We define a dominant balance regime as a region $\mathcal{R} \subset \mathcal{D}$ where the evolution equation is approximately satisfied by a subset of $p < K$ of the original terms in the equation; the remaining terms may be neglected. In this case $\mathbf{f}(x,t)$ will have near-zero entries corresponding to negligible

terms when $(x,t) \in \mathcal{R}$. Geometrically, the field is approximately restricted to p of the original K dimensions of the equation space, resulting in a subspace that is aligned with the active p terms.

This geometric perspective on dominant balance physics leads naturally to segmentation via unsupervised clustering. For example, the Gaussian mixture model (GMM) framework learns a probabilistic model by assuming the data are generated from a mixture of Gaussian distributions with different means and covariances [5]. The learned covariances for each cluster can then be interpreted in terms of active and inactive terms in the evolution equation. The N spacetime points in $\mathcal{D}$ are used to train a mixture model; the algorithm treats points from a dominant balance regime as if they were generated from a distribution with near-zero variance in the directions corresponding to negligible terms. Data beyond the original inputs can efficiently be assigned to a balance model using the trained GMM.

In practice, there is no reason to expect the points will even approximate a mixture of Gaussian distributions. We therefore expect that the number of clusters required to capture all of the relevant physics will exceed the number of distinct balance regimes, resulting in redundant clusters. Furthermore, there is some ambiguity in the interpretation of "near-zero variance". We address both of these issues using sparse principal components analysis (SPCA) [6], which uses ℓ_1 regularization to extract a sparse approximation to the leading principal component. If a cluster describes a dominant balance regime, it should be well-described by its direction of maximum variance. Moreover, this leading principal component should have many near-zero entries. We apply SPCA to the set of points in each GMM cluster and take the active terms in the cluster to be those which correspond to nonzero entries in the sparse approximation to the leading principal component. The number of models can then be reduced by grouping clusters with the same set of active terms (or equivalently, the same sparsity pattern in the SPCA approximation).

Dominant balance identification can be seen as a localized active subspace analysis in equation space [7]. Rather than assuming that there is a global decomposition into approximately active and inactive subspaces, we simultaneously search for subspaces corresponding to different balance relations and the regions of the domain where the dynamics are well-described by this subspace.

Burgers' equation

For example, one of the simplest models that demonstrates dominant balance is the viscous Burgers' equation, shown in Fig. 2. Shocks form from the nonlinear advection and are dissipated by the viscous term. Away from the shock front, however, the gradients of the field are relatively weak, so viscosity does not contribute significantly to the dynamics. Figure 2 demonstrates the balance identification procedure applied to a snapshot of the viscous Burgers' equation example. Most of the field is classified into two clusters, corresponding to either no dynamics or an inviscid balance between acceleration and advection. Only a narrow slice along the shock front belongs to a cluster in which viscosity is active.

In simple cases, this two-step GMM-SPCA procedure might be replaced with a hard threshold; if a term exceeds some value ϵ it is "on". However, the proposed method offers two main advantages over thresholding. First, the idea of dominant balance has a natural geometric interpretation in equation space, thereby avoiding setting an arbitrary threshold for which diagnostics and interpretation may not be straightforward. Second, our method considers the *local, relative* importance of terms, whereas thresholding describes *global, absolute* importance. For example, this distinction is significant in multiscale systems with some background process underlying intermittent bursts of activity. The intermittency is dominated by a balance between terms which may be much larger than the background process, although the dynamics during quiescent periods would be determined primarily by the background process. In this case an absolute thresholding method would

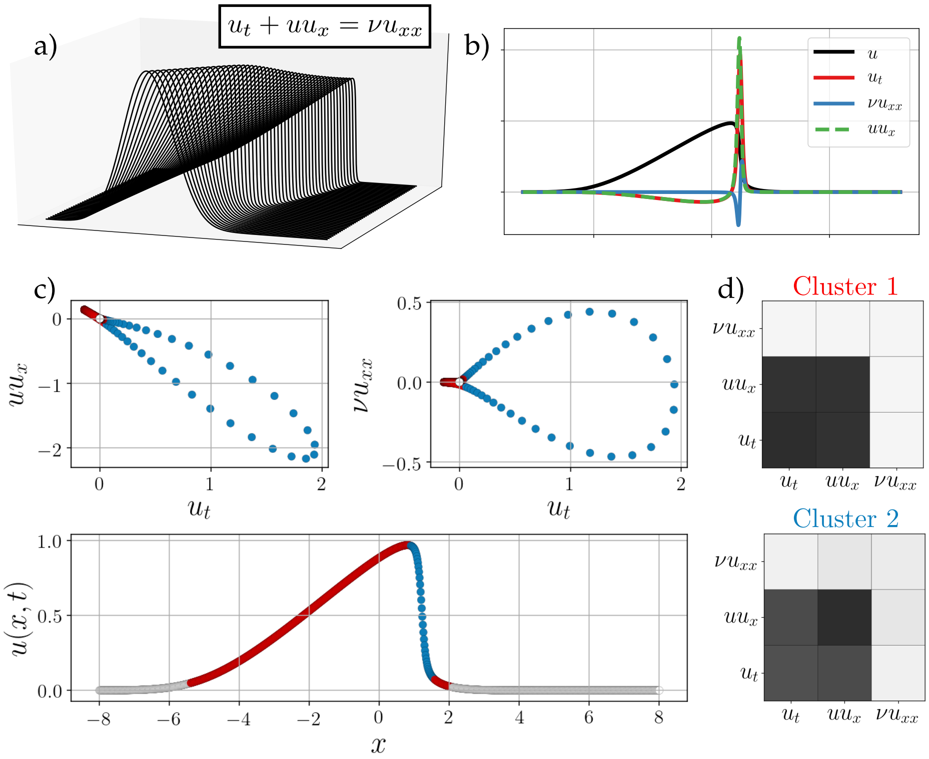

Supplmentary Figure 2: Example of dominant balance identification on the viscous Burgers' equation (a), with constituent terms shown in (b). The viscous term acts to diffuse sharp gradients and prevent formation of a discontinuous shock, but away from the shock front the dynamics are essentially inviscid. Away from the shock front, the field is approximately restricted to the $\nu u_{xx} = 0$ plane (c). This is reflected in the covariance matrices learned by the Gaussian mixture model (d).

either choose the background process to be always on or always off, whereas a relative approach recognizes that the dominant local balance simply changes during the intermittent activity. This is illustrated in Sec. , where we investigate a Hodgkin-Huxley-type model of spiking neuron, generalized to introduce multiscale bursting behavior.

Supplementary Note 2 - Results

We now apply the dominant balance identification method to a range of physics with varying complexity: unsteady vortex shedding past a cylinder at Reynolds number 100; the mean field of a turbulent boundary layer; optical pulse propagation in supercontinuum generation; geostrophy in the Gulf of Mexico; and a Hodgkin-Huxley-type model of a biological neuron. Figure 3 shows a summary of the results, including slices of the equation space representations, identified balance models, and segmented fields. In each case, the results are consistent with classical scaling analyses and known physical behavior. Descriptions of the models and code used to generate this data are presented in Appendix A and are available online.

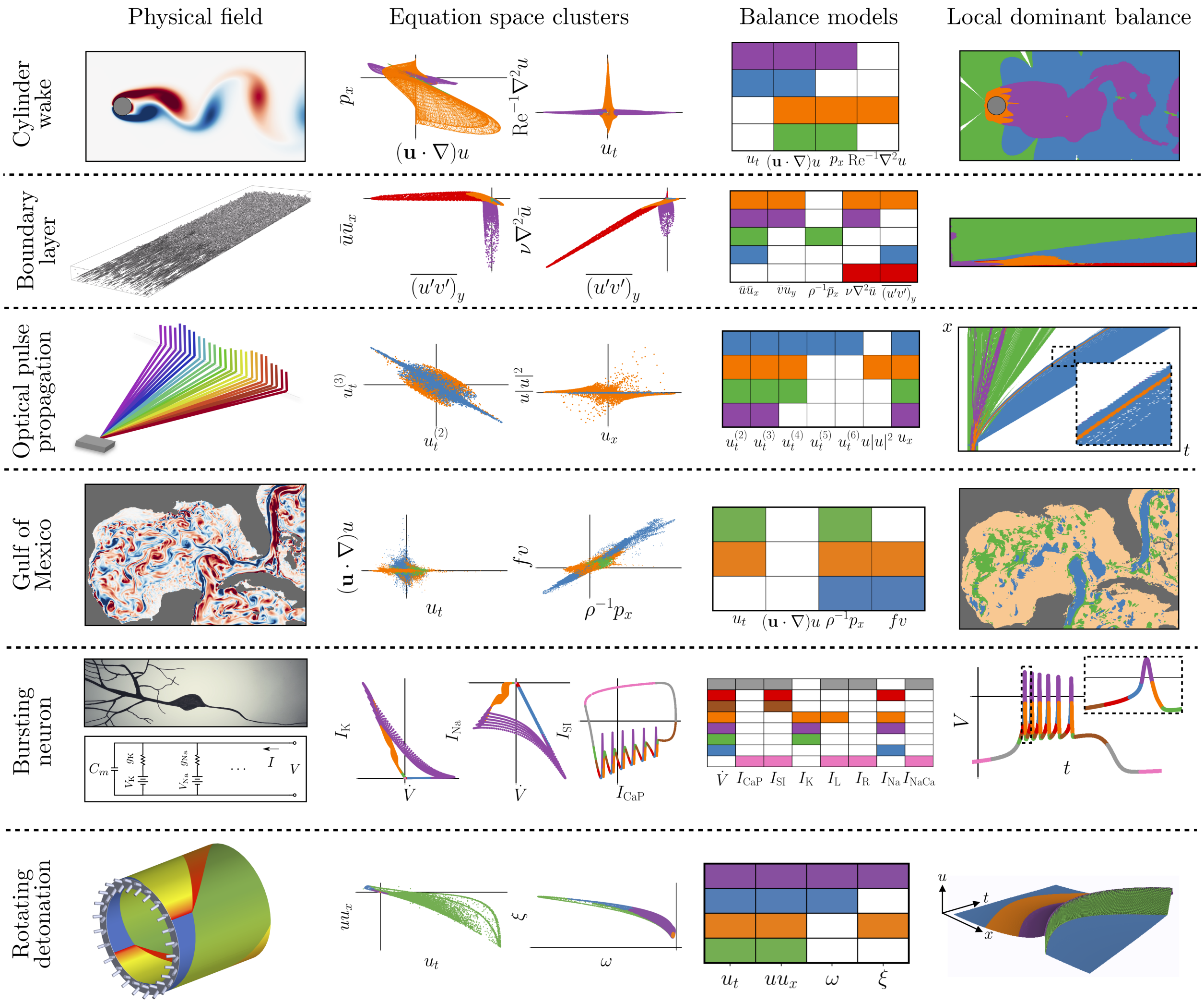

Supplmentary Figure 3: Dominant balance physics identified across a range of systems. For each case, a visualization of the system is shown on the left, followed by 2D views of the feature space colored by the identified balance relation, a key describing the active terms in each model, and the original field colored by the local balance. From top: a bluff body wake at moderate Reynolds number, a boundary layer in transition to turbulence, pulse propagation in an optical fiber, surface currents in the Gulf of Mexico, and a Hodgkins-Huxley model for an intrinsically bursting neuron.

Flow past a circular cylinder at Re $= 100$

Governing equations and analytic scaling

Flow past a cylinder at moderate Reynolds number is a prototypical flow configuration for bluff body wakes. The wake transitions from steady laminar flow to periodic vortex shedding via a Hopf bifurcation at Re $\approx$ 47. The transition from linear instability to a stable limit cycle is itself a fascinating example of dominant balance in fluid mechanics and dynamical systems. The quadratic nonlinearity, initially inactive in the linear regime, mediates energy transfer between the mean flow and instability modes, deforming both until an energy balance is reached in the periodic limit cycle. This nonlinear stability mechanism was first described by Stuart and Landau

[8, 9] and later employed for reduced-order modeling [10].

Even in the stable limit cycle, however, the local dynamics of the flow vary widely throughout the domain, highlighting mechanisms that give rise to von Kàrmàn-type vortex streets in a wide variety of flows. This unsteady, incompressible, viscous flow is governed by the two-dimensional Navier-Stokes equations:

$$\tilde{\mathbf{u}}_t + (\tilde{\mathbf{u}} \cdot \nabla)\tilde{\mathbf{u}} = -\frac{1}{\rho}\nabla \tilde{p} + \nu \nabla^2 \tilde{\mathbf{u}}, \tag{4}$$

where $\tilde{\mathbf{u}}$ is the velocity field, $\tilde{p}$ is the pressure, ρ is the density, and ν is kinematic viscosity. Of course, these equations themselves involve some degree of approximation, ignoring effects such as compressibility and gravity, making use of the Newtonian form of the stress tensor, and assuming Fickian diffusion, though they have proven highly accurate when applied in the correct regime. Nevertheless, there are distinct regimes in this simple wake flow.

For the wake behind a circular cylinder, the most relevant scales are the cylinder diameter L and free-stream velocity U. Dimensional analysis then suggests that

$$\tilde{\mathbf{u}} \sim U, \qquad \tilde{p} \sim \nu U^2, \qquad \nabla(\cdot) \sim \frac{1}{L}, \qquad \frac{\partial}{\partial t}(\cdot) \sim \frac{U}{L}.$$

Nondimensionalizing with respect to these scales, we find that the viscous term is smaller than the others by a factor of the Reynolds number, Re $= UL/\nu$, resulting in the familiar nondimensional form of the Navier-Stokes equations:

$$\mathbf{u}_t + (\mathbf{u} \cdot \nabla)\mathbf{u} = -\nabla p + \frac{1}{\text{Re}}\nabla^2 \mathbf{u}. \tag{5}$$

The variables and operators have been nondimensionalized according to the previous scales. For even moderately large Reynolds numbers, we would expect the flow to behave in an approximately inviscid manner away from the cylinder. Thus, structures formed in the near-wake region will be advected downstream by the mean flow with only weak dissipation, as observed in the vortex street.

Near the cylinder, the no-slip boundary conditions due to viscosity change the behavior qualitatively. If we examine the flow at a point a distance $\delta \ll L$ from the wall, then δ is a more appropriate length scale for the gradients. However, since the near-wall flow varies on a similar timescale to the wake, suppose that U/L is still a good scale for the time derivative. The various terms then scale as

$$\tilde{\mathbf{u}}_t \sim \frac{U^2}{L}, \qquad (\tilde{\mathbf{u}} \cdot \nabla)\tilde{\mathbf{u}} \sim \frac{U^2}{\delta} \qquad -\frac{1}{\rho}\nabla \tilde{p} \sim \frac{U^2}{\delta}, \qquad \nu \nabla^2 \tilde{\mathbf{u}} \sim \frac{\nu U}{\delta^2}.$$

We find that the acceleration term is now smaller by a factor of δ/L, and expect the viscous term to be balanced by advection and the pressure gradient. The relatively strong gradients near the wall give rise to the vortex structures which characterize the wake.

Identified dominant balance

Figure 4 shows an example vorticity field along with views of the 4D equation space corresponding to Eq. (5). Although the method treats space and time equivalently, here we freeze time and explore a single snapshot; since the flow is periodic we expect the results to be representative. The visualization in equation space clearly reveals signatures of balance relations. One set of GMM clusters is nearly restricted to the the zero-viscosity plane, while another has reduced variance

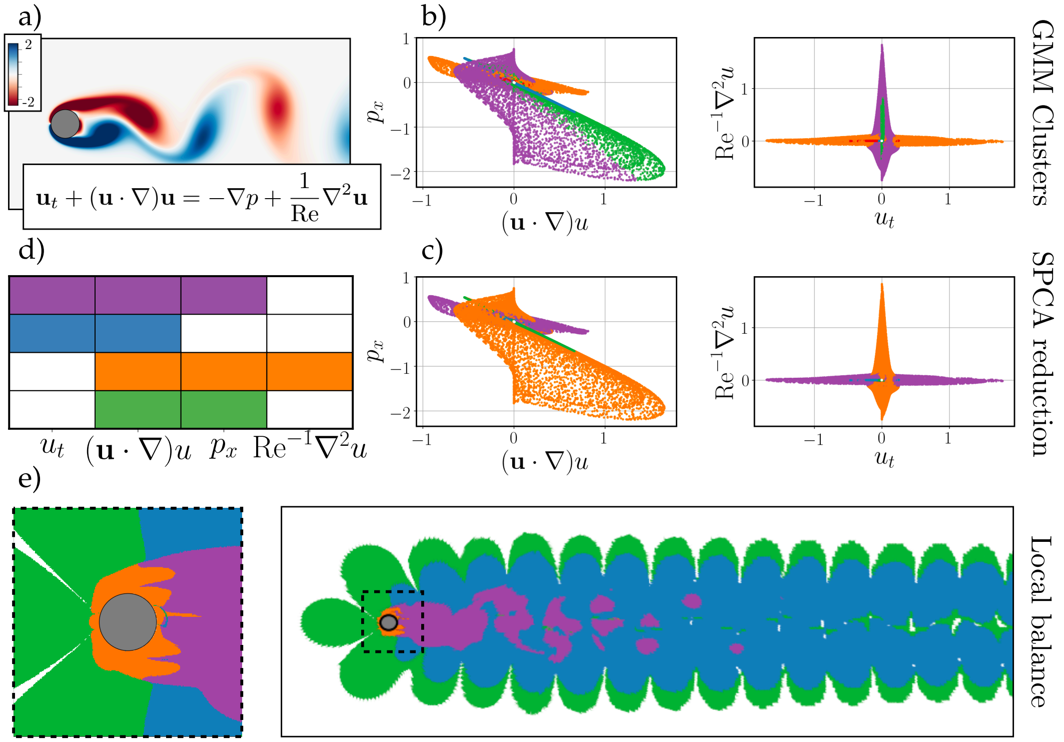

Supplmentary Figure 4: Vorticity snapshot for the wake behind a cylinder at Re $=$ 100 (a). A Gaussian mixture model (GMM) assigns field points to clusters by looking for groups with distinct mean and covariance (b). For instance, some clusters vary mainly in the acceleration-advection directions, while others vary principally in the viscous-advection directions. We would expect these to represent the far-field and boundary regions, respectively. This is confirmed by the sparse principal components analysis (SPCA) reduction, where clusters with significant nonzero variance in the same directions are grouped together (c). These directions can be interpreted as active terms in the balance relation (d). As anticipated, the region near the cylinder is dominated by a balance between viscosity and advection and pressure forces, while the far wake is approximately inviscid (e).

in the acceleration direction. The sparse approximations to the leading principal components of each cluster confirms this intuition; we use SPCA to construct balance models by grouping the Gaussian models with non-negligible variance in the same directions. As expected, the far wake is approximately inviscid, while the region near the cylinder is dominated by a balance between viscosity, pressure, and advection. This method also identifies other approximate regions, such as a low-pressure-gradient balance between acceleration and advection (blue), slowly varying potential flow (green), and a far-field region with near-zero dynamics (white).

Nonlinear stability

The cylinder wake at moderate Reynolds number is of particular interest in the reduced-order modeling community because it is a canonical example of a self-limiting instability exhibiting the Stuart-Landau nonlinear stability mechanism [10–12]. The steady-state solution $(\mathbf{U}, P)$, defined

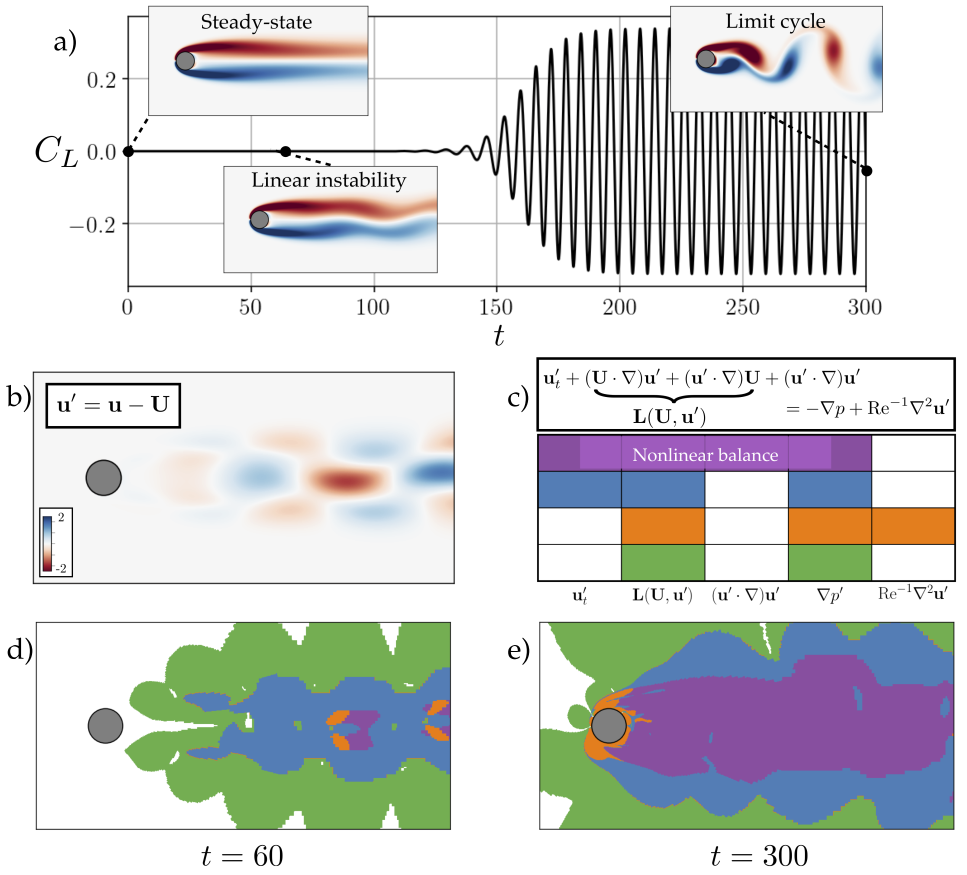

Supplmentary Figure 5: The role of nonlinearity in stabilizing a von Kàrmàn vortex street. The transient flow evolves from an unstable steady-state through the exponential growth of a linear instability mode to the vortex shedding limit cycle (a, visualized with lift coefficient C_L). Only one balance is identified that includes the nonlinear term $(\mathbf{u}'\cdot\nabla)\mathbf{u}'$. This balance does not appear significantly in the linear growth regime (d), consistent with the correspondence between the fluctuations and the instability mode. The fully saturated limit cycle is dominated by this balance, however, confirming the interpretation of the stabilizing feedback loop mediated by the nonlinearity.

as[1]

$$(\mathbf{U}\cdot\nabla)\mathbf{U} = -\nabla P + \mathrm{Re}^{-1}\nabla^2\mathbf{U}, \tag{6}$$

becomes unstable to infinitesimal perturbations at $\mathrm{Re}_c \approx 46$. At this critical parameter value, the flow undergoes a Hopf bifurcation [13, 14]; the asymptotic solution is the limit-cycle vortex shedding explored above.

The mechanism by which the exponential energetic growth of the linear instability transitions to a stable limit cycle may be understood in terms of the Stuart-Landau mean field theory. An arbitrary flow field may be decomposed into the steady-state "base" flow $\mathbf{U}$ and a time-varying perturbation $\mathbf{u}'$:

$$\mathbf{u}(\mathbf{x},t) = \mathbf{U}(\mathbf{x}) + \mathbf{u}'(\mathbf{x},t). \tag{7}$$

A similar expansion can be applied to the pressure field. Expanding the momentum equations,

[1] All fields are understood to be divergence-free; here we give only the momentum equations for brevity.

we find that the fluctuations evolve according to

$$\mathbf{u}'_t + (\mathbf{U} \cdot \nabla)\mathbf{u}' + (\mathbf{u}' \cdot \nabla)\mathbf{U} + (\mathbf{u}' \cdot \nabla)\mathbf{u}' = -\nabla p' + \mathrm{Re}^{-1}\nabla^2 \mathbf{u}'. \tag{8}$$

The only part of these equations that is nonlinear in $\mathbf{u}'$ is the advection term $(\mathbf{u}' \cdot \nabla)\mathbf{u}'$. Linear stability analysis proceeds by assuming the fluctuations are weak enough that this term is negligible.

However, the exponential growth of unstable modes implies that eventually this assumption cannot hold; in order for the energy of the flow to be bounded at long times the nonlinearity must play a stabilizing role. This is typically conceptualized as a mean field deformation. The mean of the limit cycle has a much shorter recirculation region than the steady-state solution and is approximately neutrally stable [15]. The role of the nonlinearity is thus understood as a feedback mechanism. The base flow is deformed in a manner that reduces the growth rate of the instability. The two come into energetic balance on the limit cycle, where the mean flow becomes neutrally stable; this is the basis for the "self-consistent" mean field modeling approach [12]. Although this argument is based on a linear perturbation analysis of a smoothly deforming base flow, it can be confirmed more rigorously close to the critical Reynolds number with a weakly nonlinear expansion [11].

The role of the nonlinearity can also be examined from the perspective of dominant balance, without assuming either linearity or proximity to the bifurcation. Figure 5 shows the transient evolution of the cylinder wake, from the unstable steady-state through the linear growth region and ultimately to the vortex shedding limit cycle. We apply dominant balance anlysis to the evolution equation for the base-subtracted snapshots, Eq. (8) in both the linear instability regime and the post-transient limit cycle. The method identifies four dominant balances: a far-field steady balance between base flow advection and the pressure gradient (green), a steady viscous region (orange), an inviscid linear balance (blue), and finally a balance including the nonlinear term (purple).

The nonlinear balance is largely absent from the transient growth snapshot. This is expected from the assumptions of linear stability analysis; the fluctuations at this stage are nearly identical to the linear instability mode [15]. On the other hand, the wake region of the post-transient snapshot is largely nonlinear, which confirms the picture of the role of nonlinearity in stabilizing the limit cycle.

Moreover, this analysis clearly demonstrates the instantaneous local balance of the terms, without relying on either assumptions of linearity or limiting parameter regimes. This distinguishes it from analyses such as weakly nonlinear expansions, self-consistent modeling, and energy balance arguments. Furthermore, this method is non-intrusive and could be applied to either experimental or numerical observations with more complex decomposition structures such as the harmonic balance expansion approach to nonlinear resolvent analysis [16].

Spurious terms

A key feature of the equation space representation of the evolution equation (1) is the constraint that all coordinates must sum to zero, provided the terms are computed correctly and the correct governing equation is chosen. This implies a *linear* covariance structure, even when the dynamics are strongly nonlinear; each term must be balanced by a linear combination of the others.

This constraint is not explicitly enforced in the present method, although it is the reason that the search for sparse linear subspaces with the GMM/SPCA algorithm is a natural approach. However, this observation is one avenue by which discrepancies in the governing equation may be diagnosed. If one term cannot be balanced by a linear combination of the others, it is either a spurious term or the governing equation is incomplete.

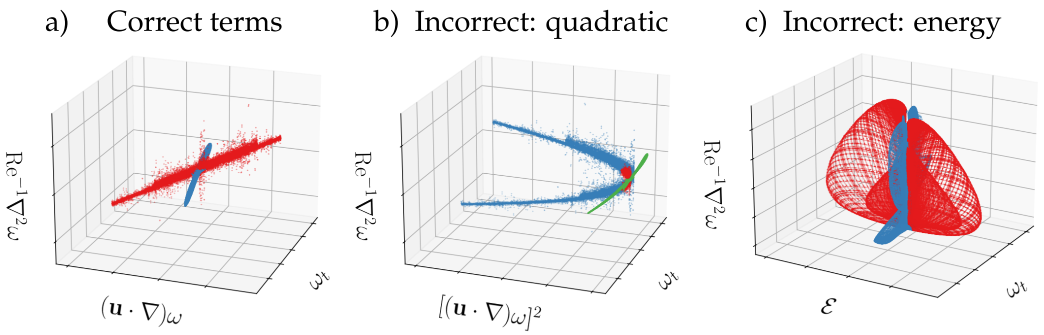

Supplmentary Figure 6: Effect of including incorrect terms in the governing equations. Even with strongly nonlinear dynamics, the fields vary along sparse linear subspaces in the equation space representation (a). By definition, the equation must be balanced by a linear combination of the terms. Introducing spurious terms such as the square of the convective term (b) or the kinetic energy (c) leads to significant departure from this behavior and may be readily diagnosed. In these cases there is either a clear nonlinear covariance structure, or the equation fails to close.

For example, in two dimensions the Navier-Stokes equations can be reduced to the form:

$$\omega_t + (\mathbf{u}\cdot\nabla)\omega = \frac{1}{\mathrm{Re}}\nabla^2\omega, \tag{9}$$

where $\omega = \nabla\times\mathbf{u}$ is the normal vorticity. As shown in Fig. 6a, the three-dimensional equation space representation has a clear linear covariance structure. One cluster (blue) has negligible viscosity and varies primarily in the unsteady-advection directions, while the other (red) is dominated by viscosity and advection, with only weak time variation.

However, if the advection term $(\mathbf{u}\cdot\nabla)\omega$ is replaced by either its square or by the total kinetic energy $\mathcal{E} = \frac{1}{2}(\mathbf{u}\cdot\mathbf{u})$, the fundamental requirement that Eq. (1) sums to zero is clearly violated, as shown by Fig. 6b-c. The clustering procedure does not give physically meaningful results, and this is easily diagnosed by considering the linear closure constraint.

Turbulent boundary layer

One of the major breakthroughs in the study of fluid mechanics in the 20th century was the development of boundary layer theory [17, 18]. In many practical applications fluids can be treated as inviscid, but close to solid boundaries strong velocity gradients lead to significant viscous forces. Prandtl showed in 1904 that careful scaling analysis applied to the governing Navier-Stokes equations reveals distinct regimes where the behavior of the fluid is essentially determined by a small subset of the full equations. In turn, these balance relations can be used to derive powerful scaling laws such as the so-called "law of the wall".

Although such analyses can be intractable for general turbulent flows, one of the most important canonical configurations is zero pressure gradient flow over a flat plate parallel to the free stream velocity. The zero pressure gradient ensures that the free-stream velocity is constant in the streamwise direction at large distances from the wall. This flow is statistically two-dimensional; the configuration does not vary in the cross-stream direction so the mean flow only varies in the streamwise and wall-normal directions.

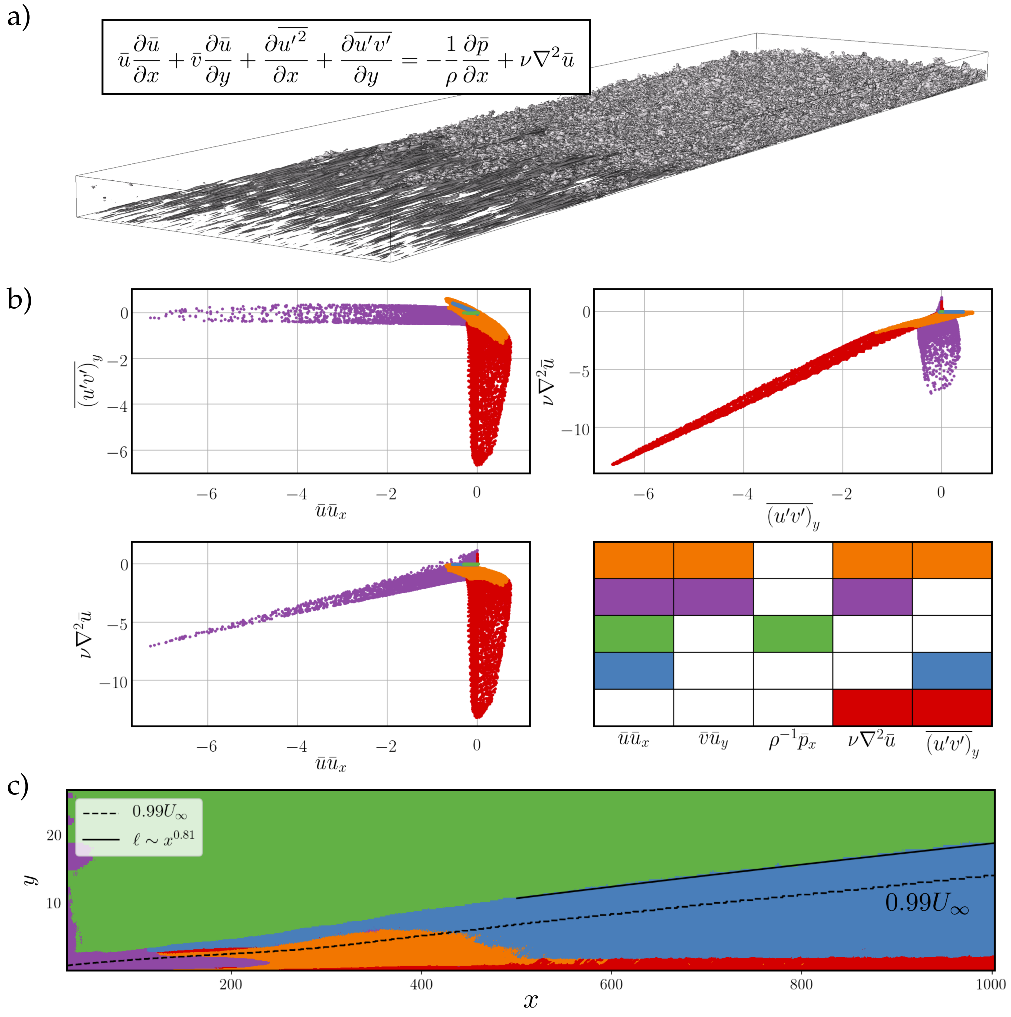

Supplmentary Figure 7: Direct numerical simulation (DNS) of a transitional boundary layer [19–23], visualized by contours of the turbulent kinetic energy (a). The Reynolds number based on free stream velocity and streamwise extent is $\mathrm{Re}_L = 192,000$. Active terms vary across the domain (b). The method recovers expected balance relations for the free-stream (green), the inertial sublayer (blue), and the viscous sublayer (red), along with a laminar region near the inlet (purple) and a transitional region (orange). The inertial sublayer follows the theoretically predicted power law (c). Boundary layer theory predicts that the length scale ℓ of the sublayer scales with $\ell \sim x^{4/5}$. As a rough criterion for the scale of the inertial balance model, we use the wall-normal coordinate at which the balance relation changes (solid line top), once the transitional region (purple) ends. A curve fit shows an approximate scaling of $\ell \sim x^{0.81}$.

Governing equations and analytic scaling

After performing the Reynolds decomposition of the variables into mean and fluctuating components, e.g. $u = \bar{u} + u'$, the mean flow is determined by the Reynolds-averaged Navier-Stokes (RANS) equations. For the streamwise mean velocity $\bar{u}$, the equation is

$$\bar{u}\frac{\partial \bar{u}}{\partial x} + \bar{v}\frac{\partial \bar{u}}{\partial y} = \rho^{-1}\frac{\partial \bar{p}}{\partial x} + \nu\nabla^2\bar{u} - \frac{\partial}{\partial y}\overline{u'v'} - \frac{\partial}{\partial x}\overline{u'^2}. \tag{10}$$

The terms on the left represent mean flow advection, while those on the right are the pressure gradient, viscosity, wall-normal Reynolds stress, and streamwise Reynolds stress, respectively.

One of the challenges in studying this flow is that there are multiple length scales. Following [24], we may consider a streamwise length scale L, a wall-normal length scale ℓ, and a viscous length scale $\eta = \nu/u_\tau$, where u_τ is the "friction velocity" associated with the shear stress at the wall.

Beginning with the "outer" region of the boundary layer (where $y \gg \eta$), suppose the mean streamwise velocity $\bar{u}$ scales with the free stream U_∞, while the turbulent fluctuations u', v' scale with u_τ. As with the previous example, assume that the derivatives scale with the corresponding length scale, so that for instance $(\cdot)_y \sim 1/\ell$. For instance, the continuity equation $\bar{u}_x + \bar{v}_y = 0$ implies that $\bar{v} \sim U_\infty(\ell/L)$. By this reasoning typically we would expect the mean velocity gradient $\bar{u}_y$ to scale with U_∞/ℓ, but as argued in [24], the gradients in the outer part of the layer are much weaker than near the wall, and empirically a better estimate is $\bar{u}_y \sim u_\tau/\ell$. Then for the streamwise momentum equation we find

$$\bar{u}\bar{u}_x \sim \frac{U_\infty^2}{L}, \quad \bar{v}\bar{u}_y \sim \frac{u_\tau U_\infty}{L} \quad \nu\bar{u}_{xx} \sim \frac{\nu U_\infty}{L^2}, \quad \nu\bar{u}_{yy} \sim \frac{\nu u_\tau}{\ell^2}, \quad (\overline{u'v'})_y \sim \frac{u_\tau^2}{\ell}, \quad (\overline{u'^2})_x \sim \frac{u_\tau^2}{L},$$

and the pressure gradient is negligible by construction. Since $L \gg \ell$ we neglect the streamwise Reynolds stress compared to the wall-normal term. On the other hand, since $U_\infty \gg u_\tau$, we can assume the mean flow advection is dominated by the streamwise component $\bar{u}\bar{u}_x$. Finally, the viscous terms are smaller than the advection by a factor on the order of the Reynolds number $\mathrm{Re}_L = U_\infty L/\nu \gg 1$. The outer part of the boundary layer is then determined by an inertial balance between streamwise mean flow advection and wall-normal Reynolds stress:

$$(\overline{u'v'})_y = -\bar{u}\bar{u}_x. \tag{11}$$

However, this relation cannot describe the near-wall regime, where viscosity is known to be important. In this region we expect the wall-normal derivatives to scale with $(\cdot)_y \sim 1/\eta = u_\tau/\nu$. As a consequence of the no-slip boundary conditions, in this region the free-stream velocity is not an appropriate scale for the streamwise component and we should instead use the friction velocity u_τ, so that

$$\bar{u}\bar{u}_x, \bar{v}\bar{u}_y \sim \frac{u_\tau^2}{L}, \quad \nu\bar{u}_{xx} \sim \left(\frac{\eta}{L}\right)\frac{u_\tau^2}{L}, \quad \nu\bar{u}_{yy} \sim \left(\frac{L}{\eta}\right)\frac{u_\tau^2}{L}, \quad (\overline{u'v'})_y \sim \left(\frac{L}{\eta}\right)\frac{u_\tau^2}{L}, \quad (\overline{u'^2})_x \sim \frac{u_\tau^2}{L}.$$

In this case the wall-normal Reynolds stress is larger than the mean flow advection by a factor of $L/\eta \gg 1$ and must instead be balanced by the viscosity. Therefore, in a thin viscous sublayer near the wall the dominant balance is

$$(\overline{u'v'})_y = \nu\bar{u}_{yy}. \tag{12}$$

The overall picture is then that the Reynolds stress must be balanced by mean flow advection in the inertial sublayer and by viscosity in the near-wall region. Outside of the turbulent boundary layer the Reynolds stresses and mean wall-normal velocity are negligible, so small variations, for instance due to incompletely converged statistics, should be described by the balance $\bar{u}\bar{u}_x = -\rho^{-1}\bar{p}_x$. In a true zero pressure gradient flow both of these would be zero in the free stream.

Identified dominant balance

We investigate the dominant balance physics of transitional boundary layer data from a direct numerical simulation [21–23], openly available from the Johns Hopkins Turbulence Database [19,

20][2]. Figure 7 shows the equation space clusters and associated dominant balance models for the mean fields. As with the cylinder example, some sets of points have significantly reduced variance in certain directions of equation space, a strong signature of the dominant balance phenomenon.

The method identifies regions corresponding to the viscous sublayer (12), inertial sublayer (11), and slightly perturbed free stream. It also identifies a region near the inlet characterized by a lack of Reynolds stresses, suggesting the mean profile here should be consistent with the laminar solution. The boundaries between balance regimes need not be sharp, however, especially in a transitional flow. In this case a cluster containing all of the active terms in the zero-pressure-gradient flat plate turbulent boundary layer equation is identified between the laminar inflow region and fully developed turbulence downstream.

Equations (11) and (12) are a starting point for many of the results of boundary layer theory; from these a range of useful laws can be derived, such as the logarithmic mean velocity profile in the inertial sublayer. Although we ultimately hope that data-driven balance identification will open new avenues of analysis, we can also use established results to examine the validity of the proposed method.

For example, the dominant length scale ℓ in the inertial sublayer is expected to depend on the streamwise coordinate x via a power law $\ell \sim x^{4/5}$ [17]. It is not usually obvious how to extract a specific value of ℓ for which this scaling can be checked. However, as a rough proxy we may consider the wall-normal coordinate at which the dominant balance changes from that of the inertial sublayer to the free-stream. Figure 7 shows the growth of the inertial sublayer thickness according to this definition along with a power law fit with exponent 0.81, showing close agreement with the expected value of 4/5. Although this evidence is somewhat circumstantial, it is at least suggestive that the balance model identification procedure reflects the underlying physics.

Self-similarity

Boundary layers are known to exhibit distinct self-similarity in both the laminar and turbulent regimes [17]. In the laminar regime, Prandtl's boundary layer equations may be solved by introducing a similarity variable $\eta = y\sqrt{U_\infty/\nu x}$ and a dimensionless streamfunction $f(\eta)$ such that $u(x,y) = U_\infty f'(\eta)$. With this ansatz the momentum equations reduce to the Blasius equation

$$2f''' + f''f = 0 \tag{13}$$

with boundary conditions $f(0) = f'(0) = 0$ and $f'(\infty) = 1$. We expect that the mean field of the transitional boundary layer will be approximately given by the Blasius solution in the regions identified with negligible Reynolds stress (green and purple in Fig. 7), which extend to approximately $x = 200$.

The left panel of Fig. 8 confirms this by comparing the numerically computed Blasius solution to the mean DNS profile. The mean flow closely matches the Blasius profile for the region identified with a laminar dominant balance (solid lines), but significant discrepancies appear in the transitional region (dotted lines).

The scaling of the turbulent region is significantly more complicated, and aspects of it are still a topic of debate [25–31]. In the inner layer, the balance between Reynolds stress and viscosity given by (12) suggests the relevant length scale is determined by the friction velocity [32]. In this case the appropriate scaling is in "wall-units":

$$y^+ = \frac{yu_\tau}{\nu} \qquad\qquad u^+ = \frac{u}{u_\tau}. \tag{14}$$

[2]https://doi.org/10.7281/T17S7KX8

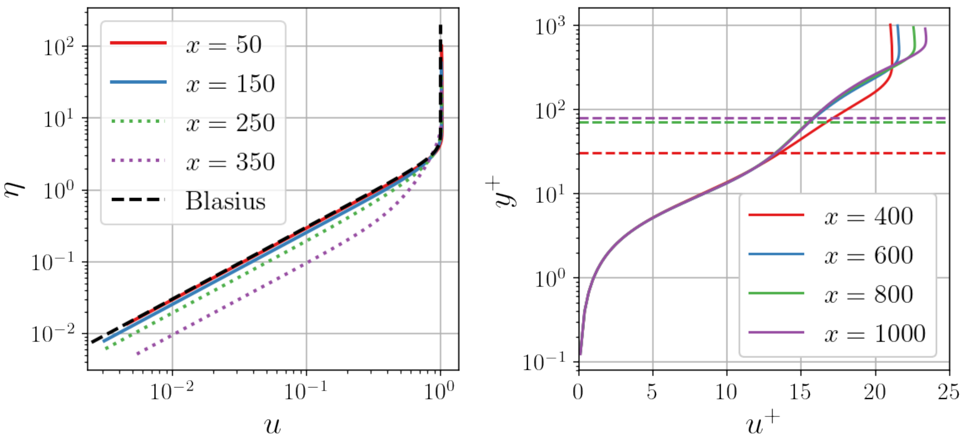

Supplmentary Figure 8: The spatial dominant balance regimes are consistent with known self-similarity in the boundary layer. The laminar balance (Fig. 7, purple and green) extends to approximately $x = 200$, a region over which the mean profile approximately matches the Blasius solution (left). Similarly, the turbulent viscous sublayer (Fig. 7, red) implies scaling with wall units (right). The mean profile with this scaling collapses until approximately the wall-normal extent of the identified viscous layer, as indicated by dashed lines.

The specific variation of u^+ with y^+ is usually throught to transition from a linear dependence in the very near-wall region to a the logarithmic "law of the wall" before the scaling changes to that of the outer inertial sublayer. Regardless of the specific functional form, the velocity profile should be self-similar in wall units throughout the viscous-dominated region (red in Fig. 7).

The right panel of Fig. 8 shows that the mean profile of the turbulent region does indeed collapse in wall units until approximately $y^+ \sim \mathcal{O}(10^2)$, as expected [32]. The dashed lines indicate the wall-normal extent of the viscous layer; the self-similarity begins to noticeably deteriorate above this point for all streamwise locations.

The identified dominant balance regions are thus consistent with expected self-similarity in the boundary layer, in both laminar and turbulent regimes. This behavior is not built into the algorithm in any way, but the result suggests that the method does indeed identify the correct physical balance in space.

Optical pulse propagation

Another important example of dominant balance arises in nonlinear optics, where the interplay of an intensity dependent index of refraction with chromatic dispersion can generate localized optical solitons [34]. The derivation of the governing evolution equations of the electric field envelope from Maxwell's equations shows that for ultra-short pulses of light (e.g. a few femtoseconds), the time response of the polarization field can yield [35] a rich set of nonlinear dynamics.

Figure 9 shows an example of a process known as supercontinuum generation, in which nonlinear processes act on a localized pulse of light to generate a severe broadening of the optical spectrum. This is typically accomplished in microstructured optical fibers [36]. Thus an initial 20-30 nanometer bandwidth can be stretched to hundreds of nanometers. The governing equation in this case is derived from Maxwell's wave equation in one dimension through the rotating wave approximation and the slowly varying envelope approximation [35]. The original PDE is linear

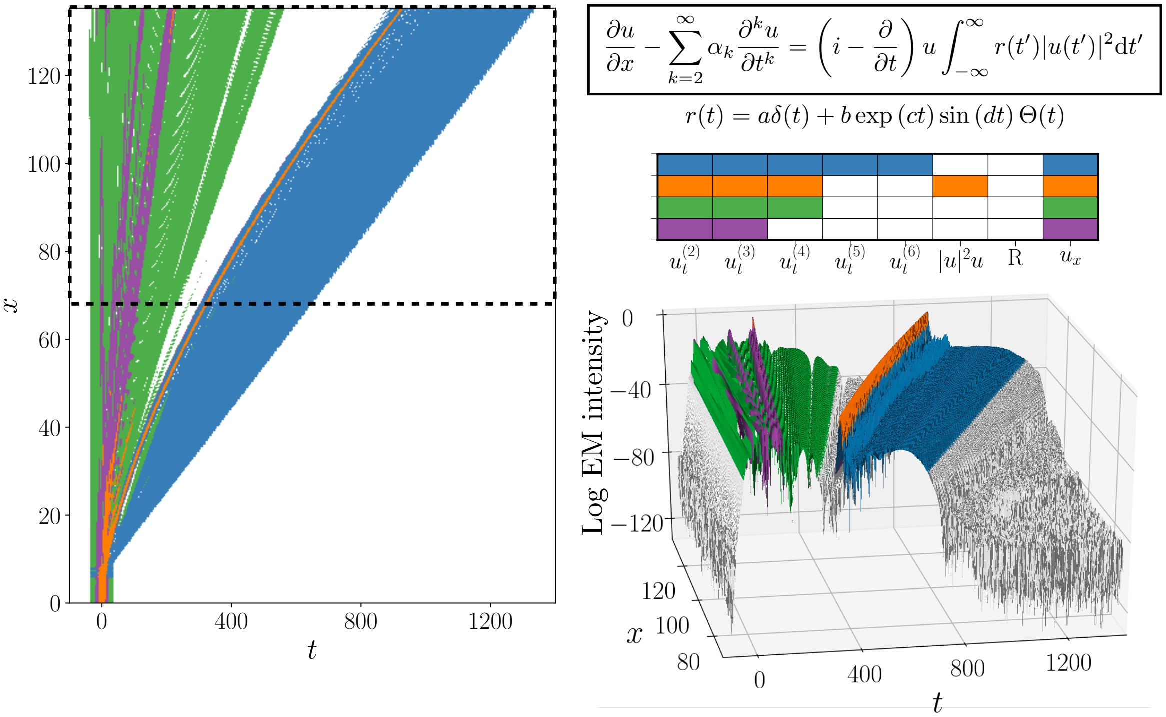

Supplmentary Figure 9: Identified balance models for the generalized nonlinear Schrödinger equation. The governing equations are derived from Maxwell's equations in 1D with a nonlinear time-delayed polarization response. Soliton propagation is understood to be maintained primarily by a balance between low-order dispersion and the cubic Kerr nonlinearity (delta-function component of the right-hand side integral) [33]. Although most of the field is identified with various linear dispersion relations, the strongest soliton is associated with cubic nonlinearity and dispersive terms through fourth order.

and second order in a vacuum, but in order to handle complicated polarization responses in fibers the field is expanded about the frequency of the original pulse [33, 37]. This "center frequency" expansion leads to a Taylor series expansion of the linear polarization response, and the Raman convolution integral describing a time-delayed nonlinear response.

Governing equation

The resulting PDE, known as a generalized nonlinear Schrödinger equation (GNLSE) describes the evolution of the slowly varying complex envelope $u(x, t)$ of the pulse. When nondimensionalized with soliton scalings [33], the envelope equation is

$$\frac{\partial u}{\partial x} - \sum_{k=2}^{\infty} \alpha_k \frac{\partial^k u}{\partial t^k} = \left(i - \frac{\partial}{\partial t}\right) u \int_{-\infty}^{\infty} r(t')|u(t')|^2 \mathrm{d}t' \tag{15a}$$

$$r(t) = a\delta(t) + b\exp(ct)\sin(dt)\Theta(t). \tag{15b}$$

The various constants (α_k, a, b, c, d) describe the polarization response and are determined empirically.

Although the spectral domain is often of practical interest for studies of supercontinuum generation, in the time domain the pulse exhibits soliton behavior, as shown in figure 9. To leading

order, the soliton propagation is typically understood to be maintained by a balance between the second order dispersion and the instantaneous part of the nonlinear response, or intensity-dependent index of refraction. That is, evaluating the delta function component of the Raman kernel leads to the cubic Kerr nonlinearity. If only this cubic nonlinearity and second order dispersion are retained, equation (15a) is reduced to the usual nonlinear Schrödinger equation (NLS):

$$i\frac{\partial u}{\partial x} + \frac{\partial^2 u}{\partial t^2} + |u|^2 u = 0. \tag{16}$$

Identified dominant balance

Figure 9 shows the balance models obtained through the unsupervised balance identification procedure applied to regions of the field where the intensity is within 40 dB of the peak. Most of the domain is associated with various linear dispersion relations, corresponding to different propagation speeds. Only a narrow region containing the strongest soliton is identified with the instantaneous nonlinear response, suggesting that a linear description is sufficient for much of the domain. The standard NLS equation is never identified, although the balance relation with cubic nonlinearity and fourth order dispersion (oran ge) is consistent with standard truncation of the linear response at third or fourth order [37]. Interestingly, the full Raman time-delay response is never selected as an important term, although this is understood to be a critical mechanism for the initial scattering. Presumably the Gaussian mixture model approach is not sensitive enough to detect this, possibly due to the clearly invalid underlying assumption of normally distributed data.

Geostrophic balance in the Gulf of Mexico

Geophysical fluid dynamics is a particularly complex field; a full description of ocean dynamics for instance requires not only the Navier-Stokes equations on a rotating Earth with complicated bathymetry, but must also account for the effects of varying salinity, temperature, and pressure via a nonlinear equation of state. The ocean dynamics also couple to atmospheric and geological processes and solar forcing [38]. Scaling analyses have been remarkably successful; despite the complexity of the dynamics, in many cases it can be argued that greatly simplified versions of the governing equations are sufficient to describe the dominant motions.

Governing equations

Perhaps the most important model of this type is geostrophic balance. To a first approximation, the surface currents can be modeled with the 2D incompressible Navier-Stokes equations on a rotating sphere:

$$u_t + (\mathbf{u}\cdot\nabla)u + fv = -\frac{1}{\rho}p_x \tag{17a}$$

$$v_t + (\mathbf{u}\cdot\nabla)v - fu = -\frac{1}{\rho}p_y, \tag{17b}$$

where ρ is the density (in general a function of temperature, pressure, and salinity), and x and y are defined in the zonal and meridional directions, respectively. The Coriolis parameter f is given in terms of the Earth's angular velocity Ω and the latitude ϕ by $f = \Omega \sin\phi$. Note that this equation already includes some approximations. Compressibility, vertical motions, and both molecular and

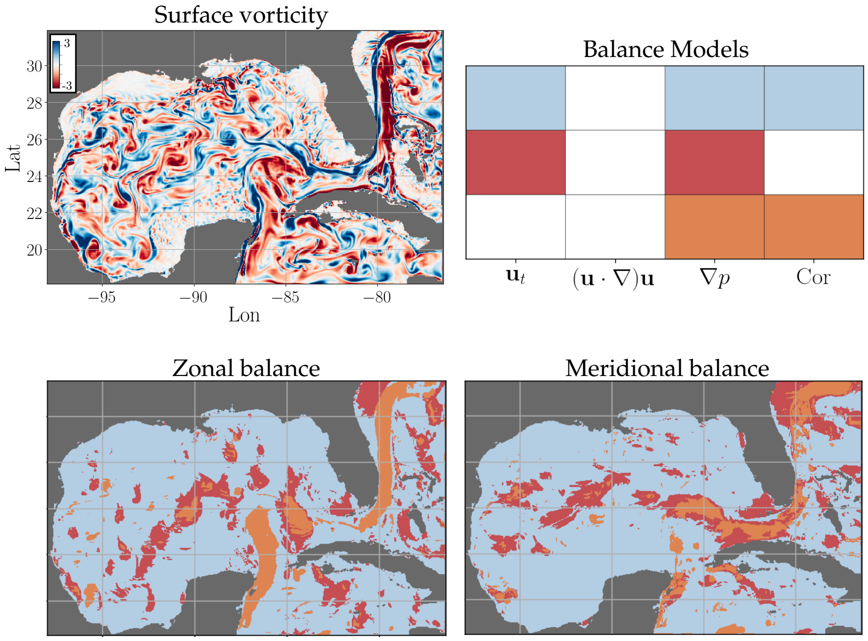

Supplmentary Figure 10: Surface vorticity in the Gulf of Mexico along with identified balance models for zonal and meridional dynamics. Orange regions are identified with the geostrophic balance, while the red regions are time-varying in response to the pressure gradient and regions in light blue are associated with the linearized rotating Navier-Stokes equations.

turbulent viscosities are all ignored in this model. Nevertheless, these equations are a standard starting point for many analyses of large scale ocean dynamics.

For flows with length scale L and velocity scale U, the relative importance of the Coriolis terms compared to the inertial terms is given by the Rossby number, $\mathrm{Ro} = U/fL$. In low Rossby number flows (relatively slow, large scale motions), the inertial terms become negligible and the dominant balance is between the Coriolis forces and pressure gradient forces:

$$+fv = -\frac{1}{\rho}p_x \tag{18a}$$

$$-fu = -\frac{1}{\rho}p_y. \tag{18b}$$

This balance is thought to describe most approximately steady large scale currents [38].

Identified dominant balance

We apply the unsupervised balance identification procedure to the high-resolution $1/25°$ HYCOM reanalysis data for the Gulf of Mexico [39]. In Fig. 10 we explore the possibility of simultaneously identifying balance regimes in multidimensional systems. Under the assumption that the same combinations of terms will appear in both zonal and meridional dynamics (but not necessarily in the same spatial regions), we compute the terms as usual and combine the equation spaces. That is, the physically equivalent terms (pressure gradient, Coriolis forces, etc) are treated identically in the clustering process. This simple approach would not generalize to multidimensional systems for which the constituent equations represent fundamentally different physics. There is no such equivalence between the conservation equations for mass and energy, for example.

Figure 10 shows the regions corresponding to balance models for this data. The method identifies three regimes; geostrophic balance (orange), a balance between acceleration and the pressure gradient (red), and the linearized rotating Navier-Stokes equations (light blue). The nonlinear advective term is not included in any of the models in this case, supporting the common use of linearized equations to study wavelike motions. Geostrophic balance is primarily identified in regions corresponding to slow, large scale motions: the southern end of the Gulf Stream and the relatively stable current between Cuba and the Yucatàn Peninsula.

Clearly the approximations in estimating gradients introduce significant error and variability into the balance identification procedure for this examples. However, the identified models are consistent with the expected behavior according to classical arguments. These results indicate some degree of robustness of the procedure and suggest that it may be applied to sufficiently clean experimental or data-assimilated observations.

Generalized Hodgkin-Huxley model of an intrinsically bursting neuron

Networks of biological neurons in an animal's nervous systems communicate with each other through the propagation of electrical potentials. These all-or-nothing events, known as *action potentials* or *spikes*, are large deviations from the membrane electrical potential at rest, as measured between the inside and outside of a neuron. Importantly, spikes can travel without significant degradation down the length of a neuron's long axon, which may be meters long.

The celebrated Hodgkin-Huxley model for spiking neurons reproduces an action potential through a balance of currents from multiple ions, each of which moves through the cell's membrane across specialized channels and pores at different phases of a spike [40]. These non-linear partial differential equations were the first detailed biophysical model to quantitively describe the dynamic activity of neurons, and they underpin decades of ongoing attempts to understand more complex properties of neuronal electrical excitability [41].

Governing equation

The propagation of an action potential along an axon is well approximated by the cable equation of a cylinder of radius a,

$$C_M \frac{\partial V}{\partial t} = \frac{a}{2r_L}\frac{\partial^2 V}{\partial x^2} + \sum_j I_j, \tag{19}$$

where C_M is the membrane capacitance, r_L is the resistivity inside the cell, and I_j are each of the ionic currents in current per unit area due to the flow of ions into and out of the cell.

Hodgkin and Huxley originally modeled three (3) ionic currents: I_{Na} sodium, I_K potassium, and a leak I_L. The dynamics of V for a single action potential can then be expressed as a system of four (4) ordinary differential equations; the balance of currents in these equations reflect the biophysical mechanisms.

Adding more ionic currents and modeling the interactive balance of their dynamics produces more complex spiking behavior. In particular, here we consider a generalized Hodgkin-Huxley model with ten (10) currents that simulates the intrinsically bursting pattern of spikes observed in the R15 neuron of the sea slug *Aplysia* [42], as shown in Fig. 11. The R15 neuron has been used to study the mechanisms underlying intrinsic bursting, where several action potentials are generated in rapid succession interspersed with relative quiet with constant inputs. Under space-clamp conditions where an entire axon cable is considered to be spatially uniform, the equation

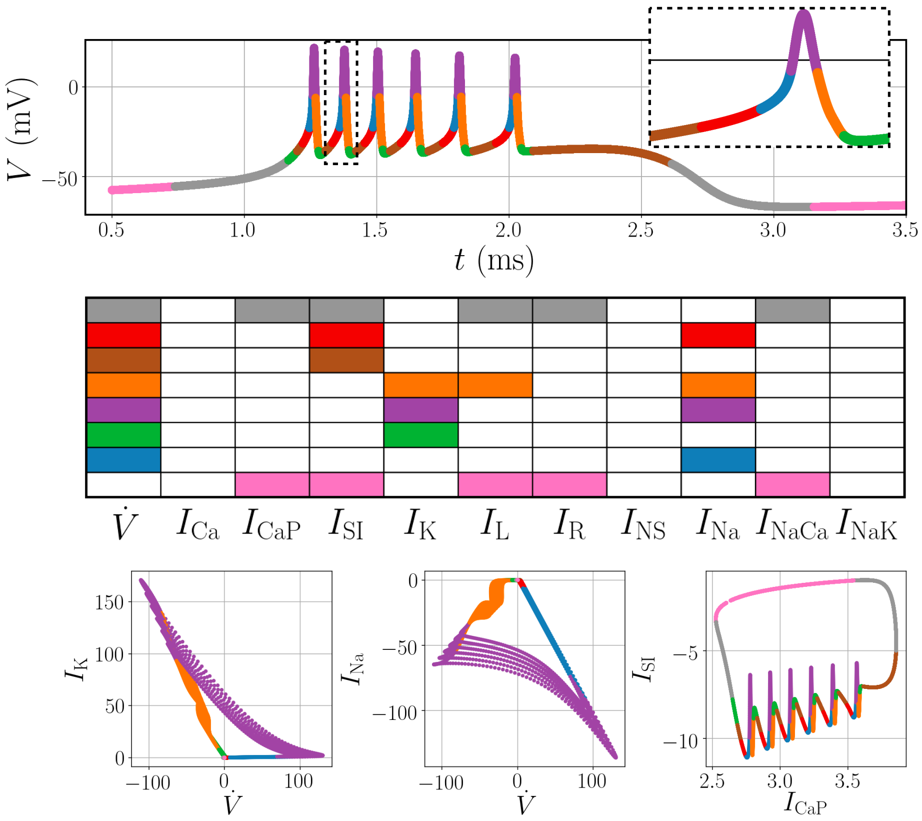

Supplmentary Figure 11: Generalized Hodgkins-Huxley model for an intrinsically bursting neuron. Dynamics in quiescent periods are characterized by currents related to calcium concentration (pink and gray), while the spiking dynamics are dominated by the classic sodium-potassium cycle.

describing the time-evolution of membrane voltage V under applied external input I_{stim} is

$$C_M \dot{V} = -\sum_j I_j + I_{\text{stim}}. \tag{20}$$

Specifically, the ionic currents I_j in our model are: I_{Na} the fast sodium Na^+ current; I_{Ca} the fast calcium Ca^{2+} current; I_K the delayed rectifier potassium current; I_{SI} the slow inward calcium current; I_{NS} the non-specific cation current; I_R the anomalous rectifier current; I_L the leakage rectifier current; I_{NaCa} the sodium-calcium exchanger current; I_{NaK} the sodium-potassium pump; I_{CaP} the calcium pump.

Identified dominant balance

Our dominant balance approach identifies several interpretable regimes of physics in the generalized Hodgkin-Huxley model that are largely consistent with known biophysics. The addition of a set of calcium-dependent currents underly the slower oscillations between quiescence and excitable bursting, as evident in the slower limit cycle. Notably, in these clusters, colored pink and gray in Fig. 11, the balance of ions is dominated by terms with strong calcium dependence (I_{CaP}, I_{SI}, and I_{NaCa}). In contrast, the time-course of V at each fast spike is dominated by voltage-gated ionic currents. In Fig. 11, the rising part of each spike is mediated by activation of sodium channels, and the inward I_{SI} and I_{Na} increase V (red and blue). V reaches peak voltage as the

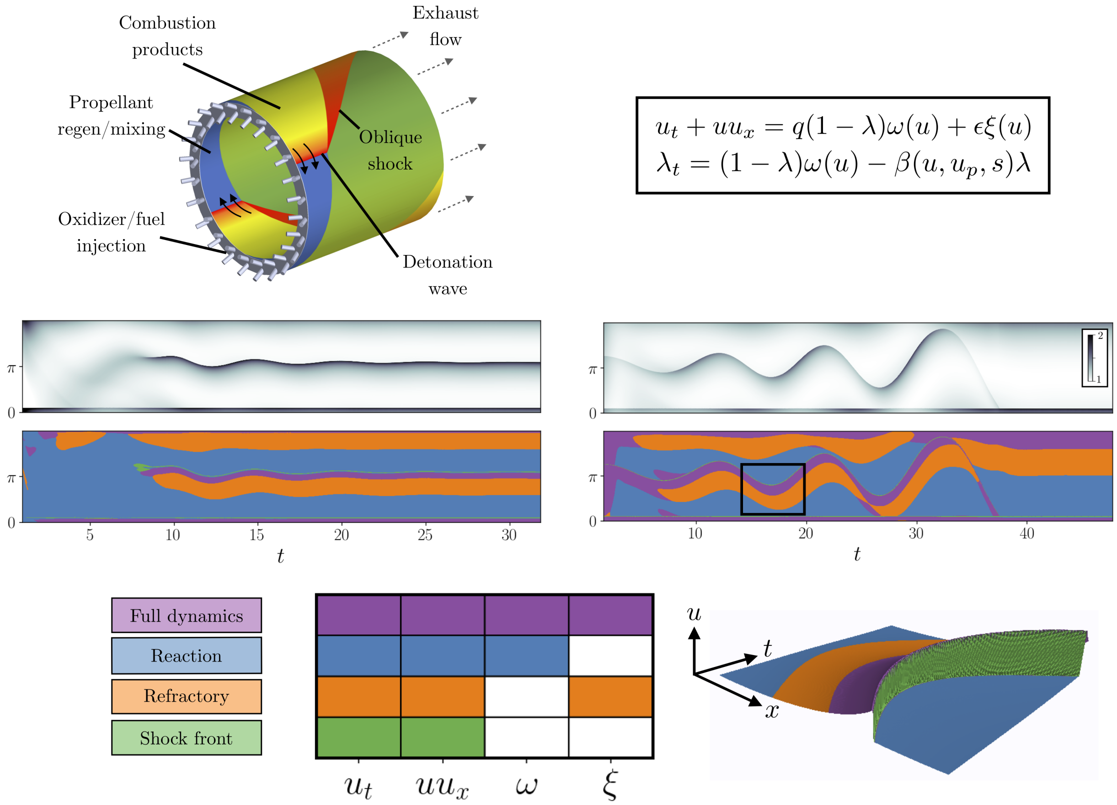

Supplmentary Figure 12: Model of combustion dynamics in a rotating detonation engine. The dynamics on the thin shock front are determined by the canonical Burgers balance (green), followed by activation of the gain and loss terms (purple). Following the combustion front, the balance transitions to the refractory exhaust-dominated period (orange). The rest of the domain is characterized by a combination of the Burgers dynamics with background energy input.

sodium channels inactivate and delayed rectifier potassium channels I_K activate (purple). The exit of potassium from the cell decreases V back towards the resting potential.

There are three currents that have not been identified to belong to any cluster: the fast calcium current, sodium-potassium pump, and the non-specific cation current. Although these are dynamically important for the model, they are relatively small compared to the other terms ($\mathcal{O}(0.1-1)$ compared to $\mathcal{O}(100)$ for the spiking dynamics) and so they don't appear to participate in any of the local dominant balance relationships identified by this method. This is a similar situation to the Raman time-delay nonlinearity in the optical pulse propagation example (Sec.) and the nonlinear advection in the Gulf of Mexico (Sec.). In all of these cases, the influence of the neglected terms appears to be of a more subtle nature than the dominant balance physics we explore in this work.

Rotating Detonation Analog System

The *Rotating Detonation Engine* (RDE) is a novel rocket engine combustor configuration that exploits the self-steepening properties of reactive compressible flows in confined, periodic geometries (such as an annular chamber, as depicted in Fig. 12) to form traveling detonation waves that persist in time [43]. For an annular RDE, fuel and oxidizer is injected at the head-end of the device, where the propellant streams rapidly mix to form a detonable mixture. Once ignited (with a spark plug, for example), the rapid exothermal chemical reaction induce gradients in temper-

ature and density within the flowfield. Because chemical kinetics are accelerated with increases in temperature, the reaction front can self steepen, eventually forming a supersonically traveling shock wave coupled to a region of rapid chemical heat release. This shock-reaction structure - the detonation wave - can travel about the annular combustion chamber so long as the ingested propellant mixture (i) contains enough chemical potential to offset dissipative effects (such as rapid expansion of the flow downstream and heat transfer to the engine walls) and (ii) is well mixed [44–47]. Thus, this combustor configuration features prominently detonative heat release - a departure from conventional constant-pressure deflagration-based engines used in aerospace applications. The potential advantages of the RDE over its deflagration-based counterparts include a potentially greater thermodynamic cycle efficiency, greater power density, and significant mechanical simplification.

The behavior of the RDE, including the development of the flowfield and the behavior of the detonation waves, is a consequence of the intricate coupling of the multi-scale physical processes present in the system [48]. The fundamental processes - injection, mixing, combustion, and exhaustion - all posses unique time and spatial scales. These range from the thickness and speed of the detonation shock front (the shortest and fastest present in the RDE system, respectively) to the length of the combustor and associated residence time of a fluid particle (the longest scales present). However, despite these scales varying by several orders of magnitude, they are intimately coupled: behind the detonation wave, the combustion products must be ejected within the period of the wave. Similarly, new propellant must be injected and sufficiently mixed prior to wave arrival. By varying the associated scales of these physics, a variety of operating modes can be obtained, including various wave counts, directions, speeds, and pattern formation with counter-propagating waves.

Phenomenological Model

A phenomenological model was recently proposed [48] to relate the associated scales of the fundamental processes of the RDE. The model adapts the inviscid Burgers' equation to a periodic domain with imposed gain (energy input from combustion) and dissipation (exhaust processes). The evolution of a representative quantity $u(x,t)$ is supplemented with a evolution equation for a combustion progress variable, $\lambda(x,t)$, which describes the balance of gain depletion and gain recovery. The model is given as:

$$\begin{aligned} u_t + uu_x &= q(1-\lambda)\omega(u) + \epsilon\xi(u) \\ \lambda_t &= (1-\lambda)\omega(u) - \beta(u, u_p, s)\lambda. \end{aligned} \tag{21}$$

where $u(x,t)$ is analogous to an intensive property of the fluid, such as internal specific energy, q is the energy release associated with the reactive mixture, $\omega(u)$ is the submodel for kinetics, ξ is the submodel for exhaust (with a loss coefficient ϵ), and $\beta(u, u_p, s)$ is the injection and mixing submodel with parameters for an injection sensitivity cuttoff u_p and overall timescale s. For the presented cases, the submodels for kinetics, dissipation, and injection are unchanged from the original presentation of the model in [48]. The model has been shows to qualitatively reproduce the nonlinear dynamics of the collection of detonation waves present in an RDE, including wave nucleation, destruction, modulation, and mode-locking.

Identified dominant balance

Two simulations of Eq. 21 are shown in Fig. 12 in the wave-attached reference frame, each showing a canonical bifurcation of number of detonation waves present in the system. Application of our

dominant balance method identifies four interpretable and distinct regions of physics, as shown in Fig. 12. We first examine these balances within the context of a steadily propagating wave. At the front of the wave is a thin region shaded in green. This region corresponds to the shock physics of the classic Burgers' equation. For this region specifically, $\omega(u)$ is approximately negligible, as the kinetics - an exponential function of u for this case - are slow until u can activate $\omega(u)$. Indeed, shortly behind the shock front appears a region shaded in blue that indicates appreciable energy release into the system. This region is also relatively thin: an accumulation of u inside the domain is required before the nonlinear dissipation submodel - a quadratic function of u - becomes significant. This occurs in the purple shaded region, where the rate of energy input to the system (which is now slowed because of the $(1-\lambda)$ multiplier with $1 > \lambda >> 0$) is of the same order as the dissipation term. Once $\lambda \approx 1$, energy input becomes negligible, though dissipation is still significant; this region is shaded in orange. This region constitutes the *refractory* period behind the detonation wave where u and λ approach rest values. For the presented simulations, the remainder of the domain is characterized by the balance of the nonlinearity of the medium (Burgers' flux) and background energy input.

In Fig. 12a, after an initial start-up transient for a single wave, a second wave nucleates and *mode locks* with the other established wave. This case highlights the result in a shift in balance physics. Prior to time $t = 7.5$, the background energy input occurring in the large blue region allowed for the accumulation of u inside the domain, thereby accelerating the rate of energy input. The positive feedback loop of energy accumulation and rate of energy input is unchecked until the dissipation term offsets the energy input term. Thus, at time $t = 7.5$, seen is the formation of a new shock front behind which is a coupled reaction and refractory zone. Immediately after nucleation, the asymmetry of wave positions causes an imbalance of available λ to each of the waves. For this case, after oscillations in phase difference between the waves, they mode-lock to the same velocity and separation distance.

A similar wave asymmetry is present in the simulation of Fig. 12b. Here, two waves briefly co-exist in a domain where there exists only enough energy flux to support a single wave. The initial perturbation in wave separation grows exponentially, until one wave overruns the other in the final high-amplitude oscillation of phase difference. During this period of instability, observed are similar oscillations in the thicknesses of regions of dominant balances. Perhaps more noteworthy, however, are (i) the apparent phase shift of these oscillations relative to the wave positions and (ii) the role of the refractory period in wave destruction. Each of the identified dominant balance regions similarly possesses an oscillatory thickness. Each layer has a unique phase shift behind the detonation wave to which the layer is attached. In the final oscillation of the two waves, one wave enters the refractory region of the other. The effect upon the wave is dramatic: it immediately weakens (lower amplitude and slower speed) and sheds its own refractory region. Therefore, once the opposing wave encounters this weak wave (with no significant refractory region), the weak wave is easily overtaken and destroyed. The remaining wave propagates stably after this bifurcation.

Supplementary Note 3 - Data provenance

Direct numerical simulation of flow past a circular cylinder.

We simulate this configuration at Re $= 100$ with unsteady incompressible DNS using the open source spectral element solver Nek5000 [54]. The domain consisted of 17,432 seventh order spectral elements on $x, y \in (-20, 50) \times (-20, 20)$, refined close to a cylinder of unit diameter centered

at the origin. Diffusive terms are integrated with third order backwards differentiation, while convective terms are advanced with a third order extrapolation. The results of this simulation have been validated against those of the immersed boundary projection method [55] by comparing aerodynamic coefficients and vortex shedding frequency. We extract the vorticity field and spatial terms in equation (5) directly from the solver for further analysis. Time derivatives for dominant balance identification were estimated with a second order central difference.

Direct numerical simulation of a transitional boundary layer.

To study dominant balance physics in the turbulent boundary layer, we use the transitional DNS by Lee and Zaki [21–23], openly available from the Johns Hopkins Turbulence Database [19, 20][3]. The full computational domain consists of a long flat plate with an elliptical leading edge. The extent of the domain (in units defined by the plate half-thickness) is $(x, y, z) \in (1040, 40, 240)$ with periodic boundary conditions in the spanwise (z) direction, discretized to $(Nx, Ny, Nz) = (4097, 257, 2049)$. Since the configuration of interest is a zero pressure gradient flat plate boundary layer, the DNS results are only saved once the flow passes the elliptical leading edge ($x > 30.2185$). The inflow consists of small amplitude free-stream turbulence superimposed on a uniform streamwise velocity U_∞ incident on the plate. The interactions of these perturbations with the laminar boundary layer cause a downstream transition to turbulence [21].

Since we are interested here in the mean momentum balance, we only use the 2D mean field (also available from JHTDB), which was computed from 4701 data snapshots once the flow reached a statistically stationary state. Without direct access to the gradients, we compute the constituent terms of the RANS equations with second-order accurate finite differences, as shown in Fig. 1b. Although some of these fields show small fluctuations, the overall smoothness suggests the statistics are approximately converged.

Supercontinuum generation in photonic crystal fiber.

The generalized nonlinear Schrödinger equation (GNLSE), nondimensionalized with soliton scaling [33], is given by Eq. (15a). The various constants describe the polarization response and are determined empirically. In this case we use the values described by Dudley *et al* for photonic crystal fiber [36]. We also use the split-step spectral method and initial conditions described in these works to simulate the pulse propagation[4].

Surface currents in the Gulf of Mexico.

We study the high-resolution $1/25^\circ$ HYCOM reanalysis data for the Gulf of Mexico [39]. We use data from only the first field in the data set, corresponding to January 1993. Data-assimilated fields are available for the 2D velocity components, sea surface temperature, salinity, and sea surface height; vorticity is shown in Fig. 10.

We must therefore estimate time derivatives and both velocity and pressure gradients to compute the terms in Eqns. (17a) and (17b). Since this information is not directly accessible from the model (as for the numerical examples), we use finite differences to estimate the velocity derivatives. The pressure field itself is also not available; as a rough estimate we use the residuals of the left-hand side of Eqns. (17a) and (17b) in place of pressure gradients. We also assume constant density throughout the field. Finally, since this field is two-dimensional but the terms in

[3]https://doi.org/10.7281/T17S7KX8
[4]MATLAB code freely available at http://www.scgbook.info/

each evolution equation represent the same physics, we simply stack the features for each velocity component into a single $(2N \times 4)$ matrix with columns corresponding to acceleration, convection, Coriolis forces, and the pressure gradient. Although these are strong assumptions and approximations, we would expect them to only make the dominant balance identification problem more difficult, since they represent attempts to deal with limited information about the system.

Generalized Hodgkin-Huxley model of a bursting neuron.

A full set of model equations, including biophysical parameters, follow [42] and are given in the simulation code. Briefly, gating variables following Hodgkin-Huxley form are described by solutions to differential equations of the general form $\dot{z} = (z_\infty - z)/\tau_z$, where z_∞ are the steady-state values and τ_z are the time constants associated with the gating variable z. To produce the data used in our analysis, this system of ordinary differential equations was integrated numerically in MATLAB using ode15.

Rotating Detonation Analog

The system was simulated using the open-source PyClaw package for hyperbolic eqations with the same parameters as [48]. The governing equations are

$$u_t + uu_x = q(1-\lambda)\omega(u) + \epsilon\xi(u) \tag{22a}$$

$$\lambda_t = (1-\lambda)\omega(u) - \beta(u)\lambda. \tag{22b}$$

Following the original paper, we use

$$\xi(u) = u^2 \tag{23a}$$

$$\omega(u) = \exp\left[\frac{u-u_c}{\alpha}\right] \tag{23b}$$

$$\beta(u) = \frac{s}{(1+e^{k(u-u_p)}} \tag{23c}$$

with parameters as follows:

q	ϵ	u_c	α	k	u_p
1.0	0.11	1.1	0.3	5	0.5

Nondimensional time τ is given in terms of the Chapman-Jouguet wave speed and domain size, in this case $\tau = t/\pi$. The nucleating wave is generated with an initial condition of $u(x,0) = 1.5\,\mathrm{sech}^1\,0(x)$ and $s = 3.5$, while the annihilating waves are generated from an initial condition of two sech pulses and $s = 2$.

Supplementary Note 4 - Parameter tuning

The proposed method was designed to minimize the number of hyper-parameters that need to be tuned. However, there are two important parameters that must be selected: the number of clusters for the Gaussian mixture model (GMM), and the ℓ_1 regularization for sparse principal components analysis (SPCA).

Since the data is not actually drawn from a mixture of Gaussian distributions it can be difficult to make a principled choice for the number of GMM clusters. Intuitively, if there are too few

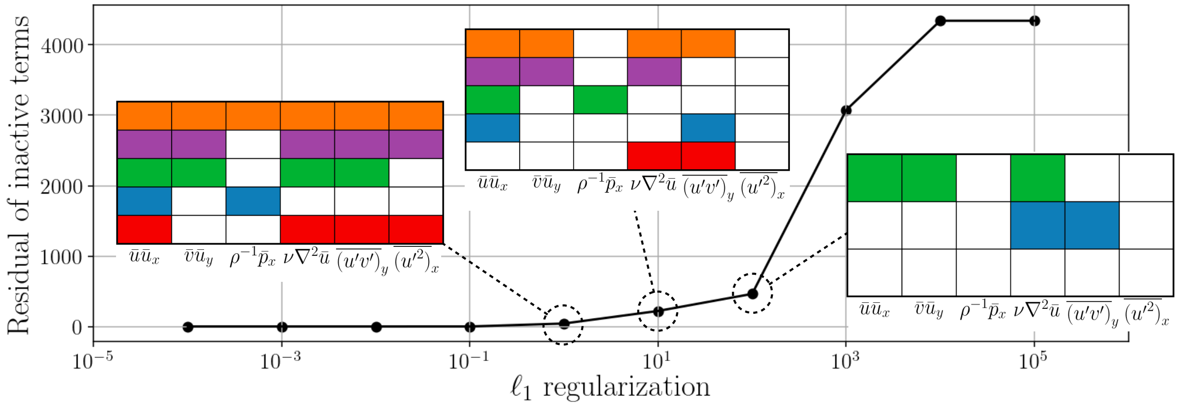

Supplmentary Figure 13: Model selection procedure used to choose a sparse regularization value for the principal components analysis, demonstrated on the turbulent boundary layer example. Although there is some flexibility depending on the desired accuracy and simplicity in the specific application, the residual of neglected terms suggests a range of appropriate values. In this work we chose regularizations that were as sparse as possible but spanned most of the original terms in the equation and had relatively small residuals (middle panel). Often this led to a set of balance relations, each with 2-3 terms, which collectively captured much of the richness of the full system.

clusters the GMM procedure cannot be expected to capture all of the distinct directions of variance in the equation space. The secondary SPCA reduction makes the method somewhat robust to this parameter; the final balance models tend to be similar provided that there are enough clusters. However, if there are too many clusters, the constituent distributions of the mixture model may not contain enough points to be dominated by a single principal component.

The ℓ_1-regularization for SPCA is somewhat easier to choose with a simple model selection procedure. A larger regularization value tends to yield more sparsity in the leading principal component, corresponding to neglected terms in the cluster. We define the residual for a given regularization value as the ℓ_2-norm of the neglected terms across all clusters. For example, if SPCA with a regularization of 0.1 yields a principal component with a zero in the direction corresponding to viscosity for one of the clusters, the SPCA residual for 0.1 in that cluster would be the magnitude of the viscous terms in that cluster. Sweeping a range of regularization values yields a Pareto-type curve showing the tradeoff of sparsity against descriptiveness.

This metric offers a guideline for choosing an appropriate regularization, although there is still some flexibility in the specific value. As Fig. 13 shows, tuning the regularization differently yields a different set of balance models. As with many model selection procedures, a different value may be selected depending on the desired level of descriptiveness and parsimony. Based on physical considerations, in this work we looked for regularizations that resulted in a diversity of balance relations with 2-3 active terms each (middle panel of Fig. 13).

Supplementary Note 5 - Model uncertainty

The idea of dominant balance is not necessarily clearly defined outside of asymptotic regimes; strictly speaking, all terms in a model are likely to have some nonzero contribution throughout the domain of interest. Considering for example the cylinder wake, clearly the boundary layer is not steady, nor is the far-field region actually inviscid.

Fortunately, since GMM is a probabilistic clustering method it comes with a natural notion of uncertainty. The clustering procedure assigns to each point a probability of belonging to each

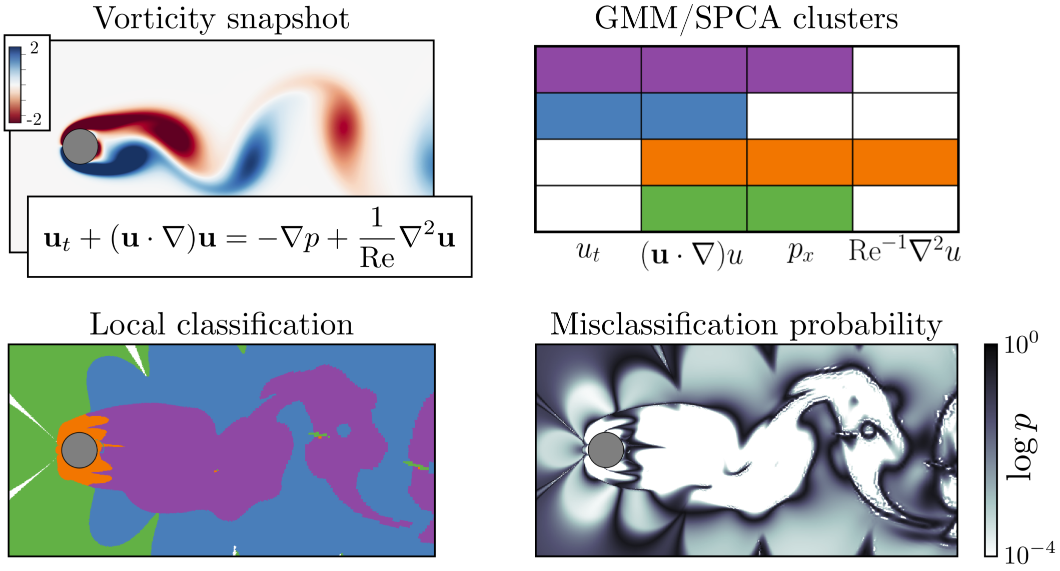

Supplmentary Figure 14: Uncertainty estimation for the dominant balance identification procedure. The Gaussian mixture model clusters points in the domain by assigning a probability of belonging to each Gaussian distribution. Summing the probabilities that each point belongs to a GMM cluster which SPCA reduces to the same balance model gives an overall estimate of the uncertainty associated with the identified dominant balance.

cluster. We can propagate this through the SPCA reduction by summing the probabilities that each point in the field belongs to one of the clusters that reduces to the same balance model. This results in an estimate of the probability of misclassification of each point, as shown in Fig. 14. As expected, this measure generally becomes large in transitional regions. However, keeping their approximate nature in mind, the balance models offer a principled and intuitive segmentation of the domain according to the dominant physics.

Supplementary Discussion

In one guise or another, dominant balance analysis has played a major role in the development of our understanding of many complex systems. In this paper we have proposed a method of identifying dominant balance regimes in an unsupervised manner directly from data. This approach leverages our understanding of the full physical complexity in the form of governing equations, but by using simple clustering and sparse approximation methods we avoid any *a priori* assumptions about balance relations. Nevertheless, in contexts ranging from fluid turbulence to nonlinear optics the method recovers classical dominant balance relationships.

The critical step in this process is the "equation space" perspective described in Sec. . By considering each term in the governing equation to describe a direction in this space, the dominant balance relations naturally manifest via restriction to sparse subspaces, i.e. dramatic reductions in variance in directions corresponding to negligible terms. This enables the Gaussian mixture models to identify clusters with variance in different directions, and the sparse principal components analysis to extract sparse subspaces by finding directions with significantly nonzero variance. These machine learning tools are therefore applied in a targeted and clearly motivated context, but the equation space perspective necessarily ties the output to underlying physics.

The method as presented here is perhaps the simplest version possible of this type of analysis. As such, there are clear opportunities for further refinement. For example, the Gaussian mixture

model analysis is built on the assumption of normally distributed data. There is no reason to think that the equation space representation of physical fields would be normally distributed, which may limit the sensitivity of the method. Other methods such as spectral clustering or a custom, =physically motivated algorithm may be more effective at segmenting this type of data.

On the other hand, the method can be sensitive to computation of the various terms in the equation, especially gradients. When possible, the terms were extracted directly from the numerical solvers, although this may present a challenge for noisy experimental data. One way to address this could be a reanalysis-type smoothing procedure, as was used by the HYCOM group to generate the Gulf of Mexico data. Similar data-assimilation approaches have been successful at resolving mean profiles of turbulent flows from limited experimental data [49, 50].

When properly developed and validated, the ability to automatically extract balance relations from data has exciting potential applications. For instance, identifying regions of flow fields where viscosity is important could be a principled way to inform schemes such as adaptive mesh refinement [51] or hybrid turbulence modeling [52, 53]; currently regions are typically chosen using heuristics or expert knowledge. An understanding of balance relations could even potentially be used to develop novel control strategies. By designing or actuating with the goal of manipulating which regimes are active, such an approach might be used to achieve drag reduction or mixing enhancement.

More generally, dominant balance analysis has historically been a critical tool for understanding local physical behavior in complex systems. To date we have only been able to apply these methods to systems for which the governing equations are well-understood and which admit an asymptotic scaling analysis. Generalizing this analytic approach with data-driven dominant balance identification could allow application of this powerful perspective to complex geometries, non-asymptotic regimes, and even systems for which the governing equations are unknown.

However, as with all applications of machine learning and data science methods to physical systems, a critical step in application to any system will be careful validation that the balance identification procedure reproduces the expected results. The dominant balance modeling approach described here is designed to build on, rather than circumvent, physical expertise. The study of dominant balance regimes has been foundational to our understanding of many complex systems; we hope that data-driven methods can integrate with this legacy to enable even wider applicability.

Supplementary References

[1] M. Schmidt and H. Lipson. Distilling free-form natural laws from experimental data. *Science*, 324(5923):81–85, April 2009.

[2] S. L. Brunton, J. L. Proctor, and J. N. Kutz. Discovering governing equations from data by sparse identification of nonlinear dynamical systems. *Proc. Nat. Acad. Sci.*, 113(15):3932–3937, 2016.

[3] S. H. Rudy, S. L. Brunton, J. L. Proctor, and J. N. Kutz. Data-driven discovery of partial differential equations. *Science Advances*, 3(4), 2017.

[4] N. M. Mangan, J. N. Kutz, S. L. Brunton, and J. L. Proctor. Model selection for dynamical systems via sparse regression and information criteria. *Proceedings of the Royal Society A*, 473(2204), 2017.

[5] C. Bishop. *Pattern recognition and machine learning*. Springer New York, 2006.

[6] H. Zou, T. Hastie, and R. Tibshirani. Sparse principal component analysis. *Journal of Computational and Graphical Statistics*, 15(2), 2012.

[7] P. G. Constantine, E. Dow, and Q. Wang. Active subspace methods in theory and practice: applications to Kriging surfaces. *SIAM Journal on Scientific Computing*, 36(4):A1500–1524, 2014.

[8] J. T. Stuart. On the non-linear mechanics of hydrodynamic stability. *J. Fluid Mech.*, 4(1):1–21, 1958.

[9] L. D. Landau and E. M. Lifshitz. *Fluid Mechanics*. Elsevier, 1959.

[10] B. R. Noack, K. Afanasiev, M. Morzynski, G. Tadmor, and F. Thiele. A hierarchy of low-dimensional models for the transient and post-transient cylinder wake. *J. Fluid Mech.*, 497:335–363, 2003.

[11] Denis Sipp and Anton Lebedev. Global stability of base and mean flows: a general approach and its applications to cylinder and open cavity flows. *Journal of Fluid Mechanics*, 593:333–358, 2007.

[12] V. Mantič-Lugo, C. Arratia, and F. Gallaire. Self-consistent mean flow description of the nonlinear saturation of the vortex shedding in the cylinder wake. *Physical Review Letters*, 113:084501, 2014.

[13] C. P. Jackson. A finite-element study of the onset of vortex shedding in flow past variously shaped bodies. *Journal of Fluid Mechanics*, 182:23–45, 1987.

[14] M. Provansal, C. Mathis, and L. Boyer. Bénard-von Kármán instability: Transient and forced regime. *Journal of Fluid Mechanics*, 182:1–22, 1987.

[15] Dwight Barkley. Linear analysis of the cylinder wake mean flow. *Europhysics Letters*, 75(5), 2006.

[16] G. Rigas, D. Sipp, and T. Colonius. Non-linear input/output analysis: application to boundary layer transition. *arXiv:2001.09440*, 2020.

[17] H. Schlichting. *Boundary-Layer Theory*. McGraw-Hill, 1955.

[18] S. B. Pope. *Turbulent Flows*. Cambridge University Press, 2000.

[19] E. Perlman, R. Burns, Y. Li, and C. Meneveau. Data exploration of turbulence simulations using a database cluster. In *Supercomputing SC07*. IEEE, 2007.

[20] Y. Li, E. Perlman, M. Wan, Y. Yang, R. Burns, C. Meneveau, R. Burns, S. Chen, A. Szalay, and G. Eyink. A public turbulence database cluster and applications to study Lagrangian evolution of velocity increments in turbulence. *Journal of Turbulence*, 9(31), 2008.

[21] T. A. Zaki. From streaks and spots and on to turbulence: exploring the dynamics of boundary layer transition. *Flow, turbulence, and combustion*, 91(3):451–473, 2013.

[22] J. Lee and T. A. Zaki. Detection algorithm for turbulent interfaces and large-scale structures in intermittent flows. *Computers & Fluids*, 175:142–158, 2018.

[23] Z. Wu, J. Lee, C. Meneveau, and T. Zaki. Application of a self-organizing map to identify the turbulent-boundary-layer interface in a transitional flow. *Physical Review Fluids*, 4:023902, 2019.

[24] P. Holmes, J. L. Lumley, and G. Berkooz. *Turbulence, Coherent Structures, Dynamical Systems and Symmetry*. Cambridge Monographs on Mechanics, 1996.

[25] G. I. Barrenblatt, A. J. Chorin, O. H. Hald, and V. M. Prostokishin. Structure of the zero-pressure-gradient turbulent boundary layer. *Proceedings of the National Academy of Sciences*, 94:7817–7819, 1997.

[26] M. V. Zagarola and A. J. Smits. Mean-flow scaling of turbulent pipe flow. *J. Fluid Mech.*, 373:33–79, 1998.

[27] J. F. Morrison, B. J. McKeon, W. Jiang, and A. J. Smits. Scaling of the streamwise velocity component in turbulent pipe flow. *Journal of Fluid Mechanics*, 508:99–131, 2004.

[28] T. B. Nickels, I. Marusic, S. Hafez, and M. S. Chong. Evidence of the k_1^{-1} law in a high-Reynolds-number turbulent boundary layer. *Physical Review Letters*, 95:074501, 2005.

[29] I. Marusic, R. Mathis, and N. Hutchins. Predictive model for wall-bounded turbulent flow. *Science*, 329(5988):193–196, 2010.

[30] A. J. Smits, B. J. McKeon, and I. Marusic. High-Reynolds number wall turbulence. *Annual Review of Fluid Mechanics*, 43, 2011.

[31] I. Marusic, J. P. Monty, M. Hultmark, and A. J. Smits. On the logarithmic region in wall turbulence. *Journal of Fluid Mechanics*, 716:R3, 2013.

[32] J. Jiménez. Near-wall turbulence. *Physics of Fluids*, 25:101302, 2013.

[33] L. F. Mollenauer and J. P. Gordon. *Solitons in Optical Fibers: Fundamentals and Applications*. Elsevier, 2006.

[34] G. Agrawal. *Nonlinear Fiber Optics, 6th Ed.* Academic Press, 2019.

[35] J. N. Kutz and E. Farnum. Solitons and ultra-short optical waves: the short-pulse equation versus the nonlinear Schrödinger equation. *Edited by H. E. Hernández-Figueroa, E. Recami*, page 148, 2014.

[36] J. M. Dudley and J. R. Taylor. *Supercontinuum generation in optical fibers*. Cambridge, 2010.

[37] K. J. Blow and D. Wood. Theoretical description of transient stimulated Raman scattering in optical fibers. *IEEE Journal of Quantum Electronics*, 25, 1989.

[38] A. Gill. *Atmosphere-Ocean Dynamics*. Academic Press, 1982.

[39] HYCOM + NCODA global $1/25°$ reanalysis (Expt. 50.1). https://hycom.org/dataserver, 2019.

[40] Alan L Hodgkin and Andrew F Huxley. A quantitative description of membrane current and its application to conduction and excitation in nerve. *The Journal of physiology*, 117(4):500–544, 1952.

[41] G Bard Ermentrout and David H Terman. *Mathematical foundations of neuroscience*, volume 35. Springer Science & Business Media, 2010.

[42] CC Canavier, JW Clark, and JH Byrne. Simulation of the bursting activity of neuron r15 in aplysia: role of ionic currents, calcium balance, and modulatory transmitters. *J. Neurophys.*, 66(6):2107–2124, 1991.

[43] Frank K. Lu and Eric M. Braun. Rotating detonation wave propulsion: Experimental challenges, modeling, and engine concepts. *Journal of Propulsion and Power*, 30(5):1125–1142, sep 2014.

[44] Fedor A. Bykovskii, Sergey A. Zhdan, and Evgenii F. Vedernikov. Continuous spin detonations. *Journal of Propulsion and Power*, 22(6):1204–1216, nov 2006.

[45] S. A. Zhdan, F. A. Bykovskii, and E. F. Vedernikov. Mathematical modeling of a rotating detonation wave in a hydrogen-oxygen mixture. *Combustion, Explosion, and Shock Waves*, 43(4):449–459, jul 2007.

[46] Takayuki Yamada, A. Koichi Hayashi, Eisuke Yamada, Nobuyuki Tsuboi, Venkat E. Tangirala, and Toshi Fujiwara. Detonation limit thresholds in h2/o2rotating detonation engine. *Combustion Science and Technology*, 182(11-12):1901–1914, oct 2010.

[47] C. A. Nordeen, D. Schwer, F. Schauer, J. Hoke, T. Barber, and B. M. Cetegen. Role of inlet reactant mixedness on the thermodynamic performance of a rotating detonation engine. *Shock Waves*, 26(4):417–428, apr 2015.

[48] J. Koch, M. Kurosaka, C. Knowden, and J. N. Kutz. Mode-locked rotating detonation waves: Experiments and a model equation. *Physical Review E*, 101:013106, 2020.

[49] S. Symon, N. Dovetta, B. J. McKeon, D. Sipp, and P. J. Schmid. Data assimilation of mean velocity from 2D PIV measurements of flow over an idealized airfoil. *Experiments in Fluids*, 58(5), 2017.

[50] A. F. C. da Silva and T. Colonius. Ensemble-based state estimator for aerodynamic flows. *AIAA Journal*, 56(7), 2018.

[51] M. J. Berger and J. Oliger. Adaptive mesh refinement for hyperbolic partial differential equations. *Journal of Computational Physics*, 1984.

[52] P. R. Spalart. Detached-eddy simulation. *Annual Review of Fluid Mechanics*, 2009.

[53] B. Chaouat. The state of the art of hybrid RANS/LES modeling for the simulation of turbulent flows. *Flow, turbulence, and combustion*, 99(2), 2017.

[54] P. F. Fischer, J. W. Lottes, and S. G. Kerkemeir. Nek5000 web pages. http://nek5000.mcs.anl.gov, 2008.

[55] K. Taira and T. Colonius. The immersed boundary method: A projection approach. *Journal of Computational Physics*, 225:2118–2137, 2007.
